# Exploring the Antidiabetic Potential of *Salvia officinalis* Using Network Pharmacology, Molecular Docking and ADME/Drug-Likeness Predictions

**DOI:** 10.3390/plants13202892

**Published:** 2024-10-16

**Authors:** Chimaobi J. Ononamadu, Veronique Seidel

**Affiliations:** 1 Natural Products Research Laboratory, Strathclyde Institute of Pharmacy and Biomedical Sciences, University of Strathclyde, Glasgow G4 0RE, UK; ononamaducj@polac.edu.ng; 2 Natural Product Research Group, Department of Biochemistry and Forensic Science, Nigeria Police Academy, Wudil P.M.B. 3474, Kano, Nigeria

**Keywords:** antidiabetic, *Salvia officinalis*, network pharmacology, molecular docking

## Abstract

A combination of network pharmacology, molecular docking and ADME/drug-likeness predictions was employed to explore the potential of *Salvia officinalis* compounds to interact with key targets involved in the pathogenesis of T2DM. These were predicted using the SwissTargetPrediction, Similarity Ensemble Approach and BindingDB databases. Networks were constructed using the STRING online tool and Cytoscape (v.3.9.1) software. Gene Ontology (GO), Kyoto Encyclopedia of Genes and Genomes (KEGG) pathways analysis and molecular docking were performed using DAVID, SHINEGO 0.77 and MOE suite, respectively. ADME/drug-likeness parameters were computed using SwissADME and Molsoft L.L.C. The top-ranking targets were CTNNB1, JUN, ESR1, RELA, NR3C1, CREB1, PPARG, PTGS2, CYP3A4, MMP9, UGT2B7, CYP2C19, SLCO1B1, AR, CYP19A1, PARP1, CYP1A2, CYP1B1, HSD17B1, and GSK3B. Apigenin, caffeic acid, oleanolic acid, rosmarinic acid, hispidulin, and salvianolic acid B showed the highest degree of connections in the compound-target network. Gene enrichment analysis identified pathways involved in insulin resistance, adherens junctions, metabolic processes, IL-17, TNF-α, cAMP, relaxin, and AGE-RAGE in diabetic complications. Rosmarinic acid, caffeic acid, and salvianolic acid B showed the most promising interactions with PTGS2, DPP4, AMY1A, PTB1B, PPARG, GSK3B and RELA. Overall, this study enhances understanding of the antidiabetic activity of *S. officinalis* and provides further insights for future drug discovery purposes.

## 1. Introduction

Diabetes mellitus (DM), a disorder of the carbohydrate, lipid, and protein metabolism characterized by hyperglycemia and dysfunctional insulin secretion or action, has become a major global health crisis [1,2,3]. According to latest statistics, about 537 million adults aged 20–79 years are living with DM, with 75% of these cases in low- and middle-income countries. It has been predicted that these numbers may reach 643 and 783 million by 2030 and 2045, respectively [4,5]. Type-2 diabetes mellitus (T2DM) is currently the most dominant type of diabetes worldwide. It is characterized by insulin resistance, a poor ability of pancreatic β-cells to secrete insulin, and is associated with vascular complications (e.g., cardio- and cerebrovascular diseases) [3,6,7].

Many plants have been used for the management of DM in different traditional systems worldwide. These include medicinal plants and plant-based foods that are integral components of daily diets [8,9]. Such plants (over 800 reported so far) are often considered to be more affordable and present fewer side effects than conventional drugs. Interestingly, they contain compounds (phytochemicals) that can prove useful for drug discovery with numerous studies reporting the antidiabetic potential of plant extracts/phytochemicals [8,10,11,12,13,14,15].

*Salvia officinalis,* commonly known as sage, is a small perennial shrub with a characteristic aromatic smell [16]. This plant belongs to the Lamiaceae family, which includes over 900 species of economic importance [17]. Although native to the Mediterranean region and the Middle east, *S. officinalis* has naturalized throughout the world and is especially common in Europe and North America. Sage has been used for centuries for medicinal and culinary purposes. It has been reported as a traditional remedy to manage seizure, ulcers, gouts, rheumatism, diarrhea, and, interestingly, hyperglycemia [18]. Pharmacological studies have already demonstrated that sage has antioxidant [19], anti-inflammatory [20] and antimicrobial activity [21]. It has also emerged as a potential antidiabetic agent, with activity demonstrated in vitro, in vivo, and in randomized controlled trials [22,23,24,25].

The field of network pharmacology, which represents a shift from the classical ‘one-target, one drug’ model, has gained significance over the past few decades as a new paradigm in drug discovery research, particularly to explore complex diseases that originate from multi-target/gene network disruptions [26,27]. It is well-suited for understanding the biological potential of traditional herbal medicines, which contain numerous phytochemicals that can exert their effects by targeting multiple proteins/genes [28]. Such an approach has already been employed to understand the antidiabetic activity of plants [29,30,31,32,33,34,35]. In the context of *S. officinalis*, it has been used to investigate antineurodegenerative activity [36,37]. To the best of our knowledge, research examining the antidiabetic properties of *S. officinalis* using network pharmacology is still lacking. Considering DM is a complex multifactorial condition that involves the interplay of different signaling pathways and molecular targets [38,39,40], it is reasonable to assume that identifying phytochemicals that interact with multiple DM targets is a beneficial therapeutic approach to follow [41,42]. The current study used network pharmacology, coupled to molecular docking and ADME/drug-likeness predictions, to explore the interactions between *S. officinalis* compounds and T2DM targets/pathways.

## 2. Results

### 2.1. Compounds Mining and Ranking

A literature search on previous phytochemicals isolated from *S. officinalis* aerial parts identified a total of 90 compounds belonging to various chemical classes. Among these, 74 were found to meet the stipulated selection criteria and were selected for further analysis (Table S1).

### 2.2. Compounds- and Disease-Associated Targets

**Figure 1:**
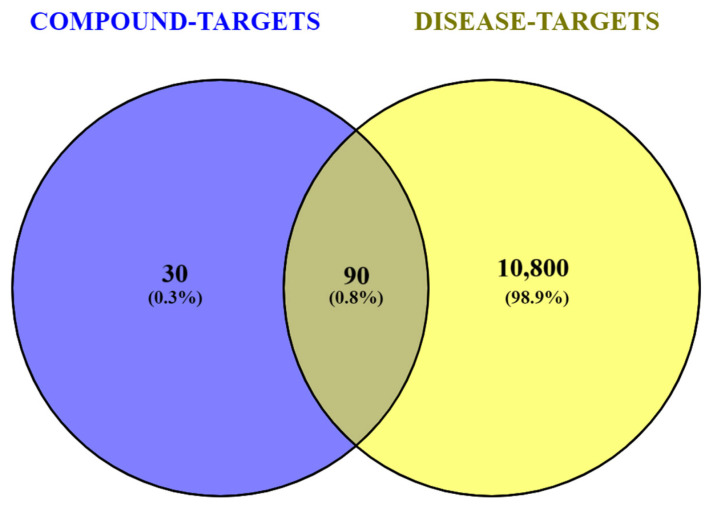
Venn diagram depiction of the biological targets common (intersection) to *S. officinalis* compounds and T2DM.

Among the selected phytochemicals, 30 were predicted to interact with 120 biological targets following uploading on the SEA, STP, and BindingDB databases. On the other hand, a comprehensive search across the GeneCards, TTD, DisGeNET, KEGG, and Malacards databases yielded 10,890 targets associated with T2DM. Only 90 targets were found to be related to both *S. officinalis* compounds and T2DM (Tables S2–S4, Figure 1).

### 2.3. Constructed Networks

#### 2.3.1. Protein–Protein Interaction (PPI) Network

**Figure 2:**
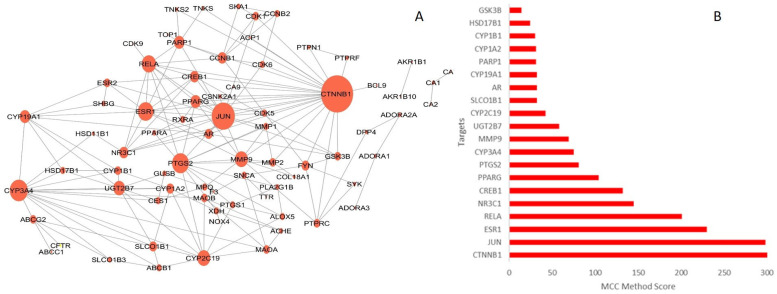
(**A**) Protein–Protein Interaction (PPI) network and (**B**) top-ranked 20 (Hub Genes) T2DM targets of *S. officinalis* compounds. The sizes of the nodes in (**A**) are proportional to the magnitude of the degree.

The protein–protein interaction (PPI) network of the targets common to *S. officinalis* compounds and T2DM was found to be significantly interconnected (confidence level of 0.7), with 75 nodes connected by 174 edges and an average number of neighbors of 4.886, indicating a moderate level of connectivity. It had a heterogeneity index of 0.856 and a centralization index of 0.300 after removing isolated nodes. The degree, betweenness centrality and closeness coefficients for all targets is presented in Table S5. The top-ranking targets (hubs) were identified as CTNNB1, JUN, ESR1, RELA, NR3C1, CREB1, PPARG, PTGS2, CYP3A4, MMP9, UGT2B7, CYP2C19, SLCO1B1, AR, CYP19A1, PARP1, CYP1A2, CYP1B1, HSD17B1, and GSK3B (Figure 2).

#### 2.3.2. Compound-Target (CT) Network

**Figure 3:**
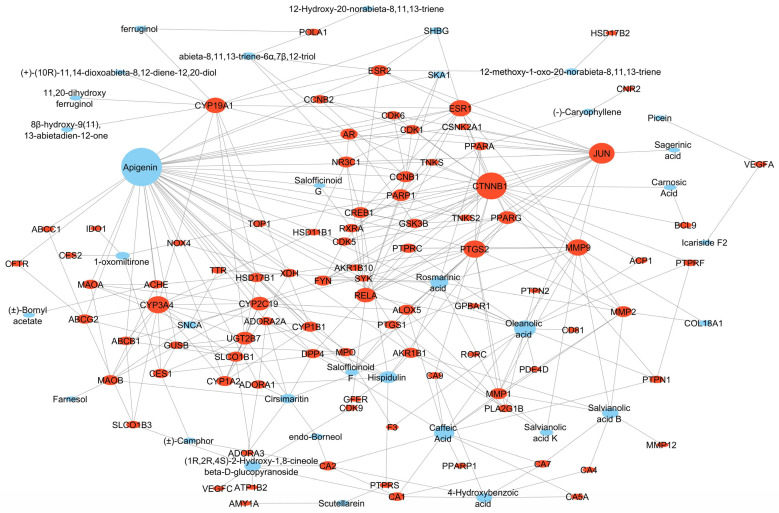
Compound-target network. The sizes of the nodes are proportional to the magnitude of the degree. Red circles = T2DM targets and blue circles = *S. officinalis* compounds.

**Figure 4:**
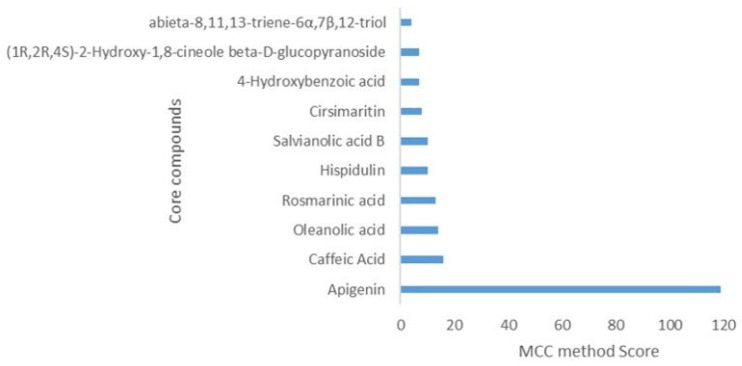
Top-ranked 10 (core) *S. officinalis* compounds predicted to interact with T2DM targets.

The 30 compounds associated with the T2DM targets were linked with the PPI network to produce a comprehensive compound-target (CT) network. The latter consisted of 125 nodes (30 compounds and 95 targets), 322 edges and an average number of neighbors of 5.246. It showed a highly connected structure of multi-targeting compounds and multi-targeted proteins. The key compounds (represented by compound nodes with a high number of connections) were identified based on the ranking of their topological parameters, with a good number of compounds showing a relatively high degree (>2). These included apigenin, caffeic acid, oleanolic acid, rosmarinic acid, hispidulin, salvianolic acid B, cirsimaritin, 4-hydroxybenzoic acid, (1*R*,2*R*,4*S*)-2-hydroxy-1,8-cineole β-*D*-glucopyranoside, and abieta-8,11,13-triene-6α,7β,12-triol with the first six compounds presenting more than five edges (connections) in the network (Figure 3 and Figure 4).

#### 2.3.3. Target-Pathways (TP) Network

**Figure 5:**
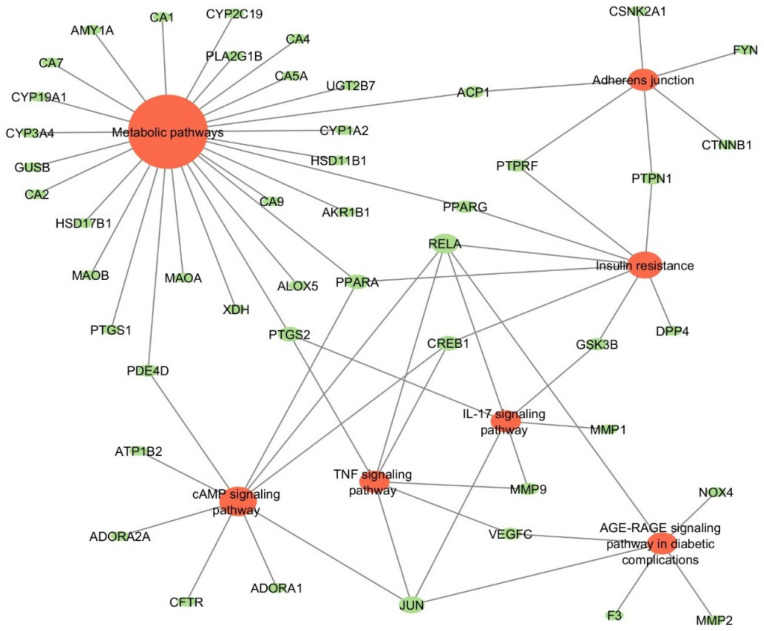
Target-pathway (TP) network of enriched T2DM-related KEGG pathways for the identified targets. Orange = KEGG Pathways, Green = Targets.

Several connections between the identified common targets and highly enriched pathways relevant to T2DM were observed. This included those involved in insulin resistance, adherens junctions, metabolic processes, and the IL-17, cAMP, relaxin, TNF signaling pathways as well as the AGE-RAGE signaling pathway in diabetic complications (Figure 5).

### 2.4. Enriched KEGG Pathwas and GO

**Figure 6:**
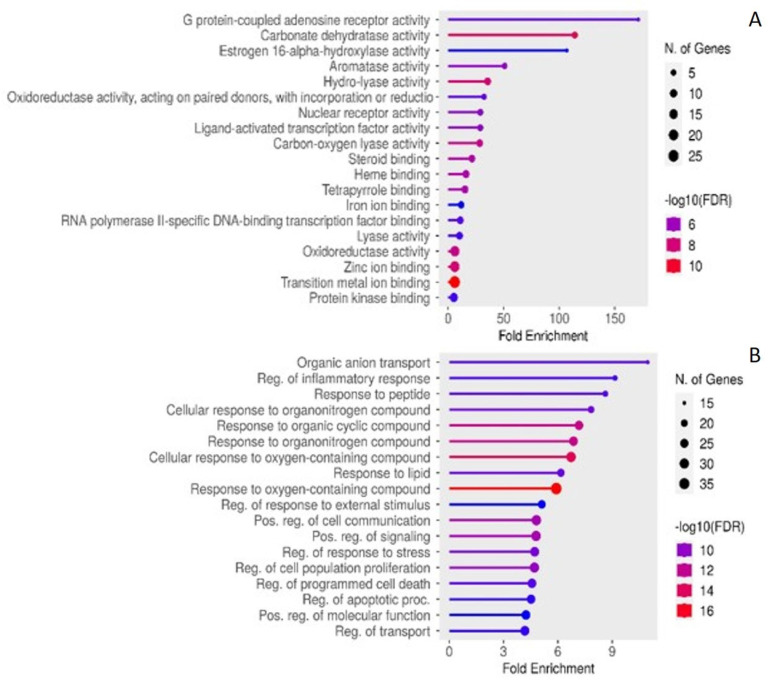
GO and KEGG pathway analysis showing the top-ranked enrichments of (**A**) molecular function and (**B**) biological process for the common targets/genes associated with *S. officinalis* compounds and T2DM.

**Figure 7:**
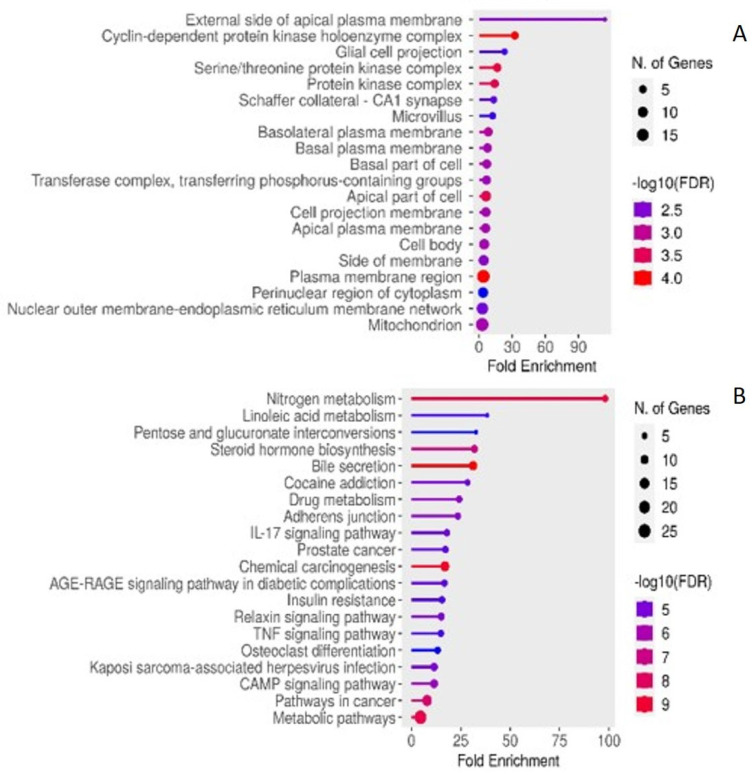
GO and KEGG pathway analysis showing the top-ranked enrichments of (**A**) cellular compartment (**B**) KEGG pathways for the common targets/genes associated with *S. officinalis* compounds and T2DM.

The enriched molecular functions, biological processes, cellular components and KEGG pathways associated with the identified common targets are listed in Figure 6 and Figure 7.

### 2.5. Molecular Docking

**Figure 8:**
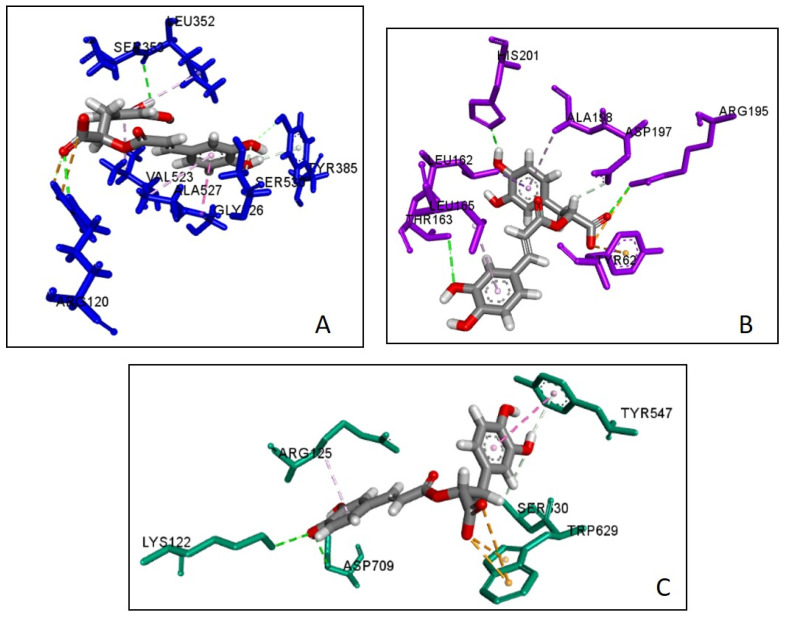
Docked pose of rosmarinic acid with (**A**) PGTS2 (**B**) AMY1A and (**C**) DDP4.

**Figure 9:**
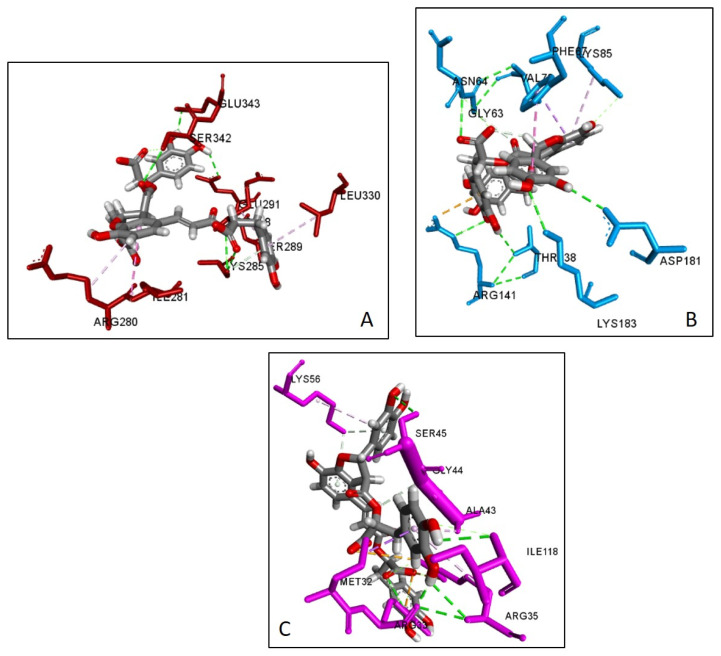
Docked pose of salvianolic acid B acid with (**A**) PPARG, (**B**) GSK3B and (**C**) RELA.

**Figure 10:**
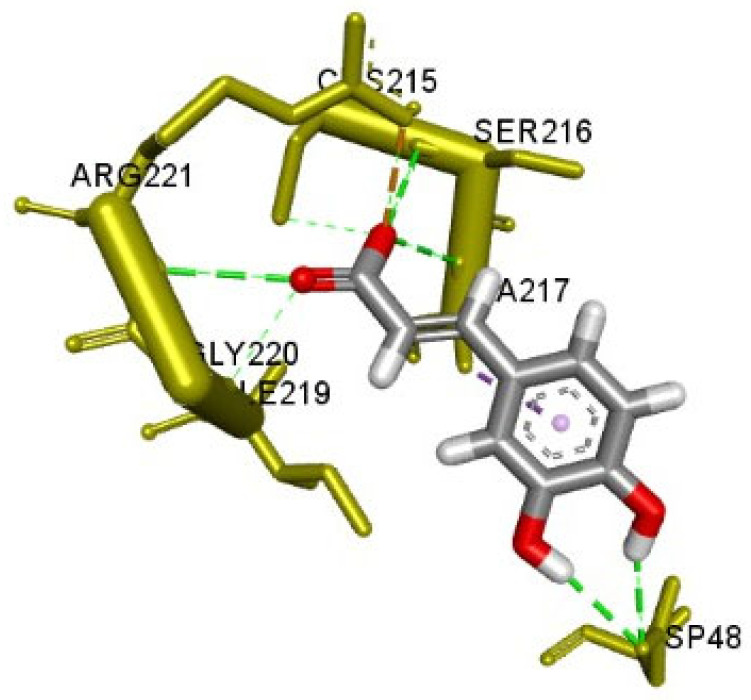
Docked pose of caffeic acid with PTP1B.

The first six compounds presenting more than five edges in the CT network (i.e., apigenin, caffeic acid, oleanolic acid, rosmarinic acid, hispidulin, salvianolic acid B) were docked with seven key targets relevant to DM, namely, PTGS2, PPARG, PTP1B, DPP4, GSK3B, AMY1A and the NF-κB p65 subunit (RELA). Rosmarinic acid showed the best SILE value amongst all ligands towards PTGS2, DPP4 and AMY1A. This value was comparable to the control ligand in the docking against PTGS2. Salvianolic acid B showed the best SILE value towards PPARG, GSK3B and RELA. This value was higher than that of the control in the docking against RELA. Caffeic acid showed the best SILE value towards PTB1B. All ligands exhibited diverse interactions with amino acid residues of the protein targets. The docking scores, SILE values and interactions predicted for each ligand are presented in Table 1, Table 2, Table 3, Table 4, Table 5, Table 6, Table 7 and Table 8 and Figure 8, Figure 9 and Figure 10.

## 3. Discussion

Protein–protein interaction (PPI) networks are essential for understanding biological functions and mechanisms. Like most biological networks, PPIs can be characterized by their topological parameters that include degree, betweenness, and closeness centrality. This characterization helps determine the ranking of nodes to identify crucial hubs that regulate disease-associated pathways and offers insights into interactions and their functional consequences in disease conditions. Hub genes (i.e., nodes with significantly high numbers of connections) are central to these networks and play a vital role in maintaining cellular integrity and function. To date, more than half a million PPI dysregulations have been linked to pathological events. Therefore, targeting these genes has become a focus in recent times as a promising therapeutic approach for various diseases [43,44]. In the current study, the hub targets identified in the PPI network included CTNNB1, JUN, ESR1, RELA, NR3C1, CREB1, PPARG, PTGS2, CYP3A4, MMP9, UGT2B7, CYP2C19, SLCO1B1, AR, CYP19A1, PARP1, CYP1A2, CYP1B1, HSD17B1, and GSK3B.

To understand how these targets (proteins/genes) are implicated in the molecular mechanisms of T2DM and the reported antidiabetic activity of *S. officinalis*, a pathway enrichment analysis was performed using predicted common targets. The resulting KEGG pathway analysis identified several pathways that were related to DM as well as cancer. The DM-linked pathways identified (i.e., insulin resistance, adherens junctions, metabolic processes, and the IL-17, cAMP, relaxin, TNF, and AGE-RAGE signaling pathways involved in diabetic complications) were used to construct the target-pathway (TP) network. These pathways encompassed most of the predicted hub targets and others, including RELA (NF-κB (P65)), JUN, PTGS2, CREB1, PPARG, PPARA, ACP1, MMP9, PTPRF, PTPN1, PDE4D, GSK3B, VEGFC, DPP IV, AMY1A, and NOX4. Among these, PPARγ, DPP IV, and AMY1A are known targets for the current antidiabetic drugs thiazolidinediones, dipeptidyl peptidase IV (DPP-4) inhibitors or gliptins, and α-glucosidase inhibitors, respectively [45]. Others, including RELA, PTGS2, CREB1 and GSK3B, have been reported as promising targets for novel antidiabetic drugs.

RELA, JUN, and PTGS2 mediate inflammatory pathways that ultimately drive insulin resistance, diabetes, and its complications. RELA (p65) is a protein that belongs to the NF-κB transcription factor family which also consists of REL (c-Rel) and NFKB1 (p105) activated by the canonical signaling pathway, along with RELB (RelB) and NFKB2 (p100) activated by the non-canonical pathway. These proteins regulate various cellular processes including inflammation, tumor necrosis factor (TNF-α, PGTS2 expression [46] as well as the immune response implicated in DM [47,48,49]. RELA is essential for maintaining normal glucose homeostasis and regulating pancreatic islet enhancer hubs [49]. In a high-fat diet L-p65 knockout mouse model, there was a marked improvement in systemic and hepatic insulin sensitivity, along with suppression of cAMP/PKA signaling [47]. The c-Jun protein (JUN) on the other hand is a transcription factor that forms various complexes collectively termed the activation protein-1 (AP-1). It is implicated in the transcription and expression of pro-inflammatory mediators, ultimately driving insulin resistance in adipose tissue [50]. Although there are no reports of a direct implication of the c-Jun protein in T2DM therapeutics, the activating kinase, c-Jun N-terminal kinase (JNK), has been linked to insulin resistance, dysfunctional β-cells, and the transition from obesity to DM [51]. Experimental studies using diabetic animal models have indicated that the inhibition of JNK can improve insulin resistance [52].

Prostaglandin endoperoxide synthases (PTGS) are crucial in various patho/physiological processes, notably, the inflammatory and immune response, tumor growth, renal injuries and cell homeostasis [53]. Although a link between the expression of PTGS2 and diabetogenic events has been established, the mechanisms involved are still poorly understood [54,55]. It has been demonstrated that PTGS2 inhibitors improve insulin sensitivity in overweight and obese human subjects [56]. Although, aspirin has been shown to normalize hyperglycemia in T2DM, the direct involvement of PTGS2 as a target for DM is still debatable [57].

Cyclic adenosine monophosphate (cAMP) response element-binding protein 1 (CREB1), a member of the cAMP-activating transcription factor family plays an important role in gluconeogenesis, lipid metabolism, and insulin signaling via the cAMP/PKA pathway [58]. Functional polymorphisms in CREB1 associated with T2DM has been reported in the Chinese population [59]. Targeting CREB-1 has demonstrated promising antidiabetic potential, by reducing fasting plasma glucose, cholesterol, and triglycerides levels in an animal model of T2DM [60].

Glycogen synthase kinase 3 (GSK-3) belongs to a highly conserved family of serine/threonine protein kinases involved in the regulation of glycogen metabolism and various other regulatory activities [61]. Dysregulation of GSK-3β is associated with insulin resistance. Consequently, inhibitors of GSK-3 have been explored for their antidiabetic effects [61,62,63]. Additionally, GSK-3β has been reported to be a common link between T2DM and Alzheimer’s disease [64].

Mechanistically, it can be inferred that the components of *S. officinalis* exert their effects by interacting with specific targets that drive the pathways enriched in the TP network. Insulin resistance, along with certain inflammatory signaling pathways, plays a major central role in this network, indicating the importance of these pathways in the molecular mechanism of *S. officinalis* compounds. From the TP pathway, the insulin resistance signaling pathway linked up with other pathways via the targets discussed thus far. This included metabolic pathways (via PPARα, PPARγ), cAMP-signaling (via RELA), inflammatory and immunological pathways like IL-17 (via GSK-B, RELA), TNF-α (via CREB1, RELA), AGE-RAGE in diabetic complications signaling (via RELA) and adherens junctions (via PTP1N, PTPRF).

Insulin resistance is often associated with obesity, which triggers a low-grade inflammatory response involving pro-inflammatory mediators such as TNF-α, IL-17, and NF-κB [65,66,67]. This inflammatory response, along with insulin resistance, is linked to the development, progression and complications of T2DM [68]. Hence, this serves as an important point of focus in studying diabetes therapeutics. IL-17α is a pro-inflammatory cytokine that can activate the NF-κB pathway and increase the release of other pro-inflammatory mediators (TNF-α, IL1-β, IFN-γ) as well as enzymes such as inducible nitric oxide synthase (iNOS) [68,69], cyclooxygenase (COX)-2, and matrix metalloproteinases (MMPs) [70]. TNF-α, on the other hand, activates the NF-κB and AP-1 pathways, which are also essential for the inflammatory response [71]. The direct implication of TNF-α in inducing insulin resistance in different tissues (skeletal and adipose) has been well documented [72,73,74], with TNF-α suppression leading to improved insulin sensitivity in vivo [73].

Adherens junctions provide essential adhesive contact support between neighboring endothelial cells [75]. In DM, the cadherin-mediated function of adherens junctions in insulin secretion by pancreatic β-cells is disrupted [76,77].

cAMP is an intracellular secondary messenger that plays an important role in regulating metabolism and glycemic homeostasis [78]. It is critical for insulin secretion by pancreatic β-cells [79] and activated by glucagon to promote the expression of key enzymes required for gluconeogenesis in hepatocytes via phosphorylation of cyclic AMP-responsive element-binding protein 1 (CREB-1) in a pathway involving protein kinase A (PKA) [80]. The cAMP/PKA signaling pathway has been identified as a target for antidiabetic drugs like glucagon-like peptide 1 (GLP-1) agonists and DPP 4 inhibitors that improve insulin secretion [81], as well as biguanides that suppress the glucagon-mediated cAMP production [82]. Interestingly, the phosphodiesterase IV (PDE4D) inhibitor roflumilast has been reported to ameliorate the symptoms of T2DM and insulin resistance by potentiating cAMP signaling, possibly influencing insulin secretion [83].

The hormone relaxin is structurally related to insulin and insulin-like growth factor [84]. Relaxin supplementation has been reported to reverse insulin resistance, improve glucose homeostasis and ameliorate vascular complications in DM [85].

Advanced glycation end products (AGE) that originate from the metabolic dysregulation observed in DM are able to generate reactive oxygen species (ROS) and when interacting with their receptor (RAGE) can trigger other pathways such as MAPK (ERK1/2, p38, JNK), PI3K/Akt, JAK/STAT1 that lead to vascular diabetic complications [86,87].

These results were also supported by the predicted GO terms of specific biological processes (inflammatory response, external stimulus, cell–cell communication, response to stress, cell proliferation and apoptotic process), cellular component (membrane, transcription factor complex, mitochondrion, cytosol, cytoplasm), and molecular functions (G-protein coupled receptors, oxidoreductase, protein kinases, nuclear receptor, ligand-activated transcription factor protein, steroid binding, DNA-binding transcription factor binding, and metal binding activities). The GO terms were consistent with the activities and components of the important targets and pathways predicted (e.g., the PPARs and the ligand-activated transcription factor protein).

From the results of the PPI and TP networks, it is interesting to note that some of the identified targets are known to be implicated in the pathogenesis of both T2DM and certain types of cancers. This was also confirmed following the GO and KEGG pathway enrichment analysis, as several pathways, molecular functions, biological processes, and cellular compartments associated with DM but also linked to cancer were predicted, thereby corroborating the previously reported relationship between these two conditions [88,89,90,91,92]. This suggests that, along with its antidiabetic effect, *S. officinalis* could have additional potential benefits in the management of cancer.

A compound-target (CT) network visually depicts the interactions between compounds and their molecular targets that are relevant to the pathogenicity of a given disease. This provides a thorough and holistic understanding of the potential pharmacological mechanisms of action involved. In the current study, apigenin, caffeic acid, oleanolic acid, rosmarinic acid, hispidulin, salvianolic acid B, cirsimaritin, 4-hydroxybenzoic acid, (1*R*,2*R*,4*S*)-2-hydroxy-1,8-cineole β-*D*-glucopyranoside, and abieta-8,11,13-triene-6α,7β,12-triol were identified as the top-ten *S. officinalis* compounds predicted to interact with multiple T2DM targets in the CT network. This was predicted to involve the modulation of multiple pathways important in T2DM in the TP network. It is interesting to note that the flavonoid apigenin has previously been reported to improve insulin resistance and dyslipidemia [93], potentiate incretin (GLP-1), modulate the CREB-BNDF signaling pathway, and improve cognition in high-fat diet-induced obese rats [93,94]. It has also been demonstrated to bind to PPARγ and reduce obesity-associated inflammation [95], as well as reduce pro-inflammatory cytokine production by inhibiting NF-κB and TNF-α activation in a carrageenan-induced inflammation murine model [96]. It suppressed NF-κB translocation in streptozocin-induced diabetic cardiomyopathy [97] and inhibited PTGS2 activity in tumor cells [98]. Apigenin also has DPP-4 [99], AMY1A [100] and PTP1B [101,102] in vitro inhibitory activity. This corroborates its strong connections observed in the CT network.

Hispidulin is another flavonoid which has been reported to stimulate GLP-1 secretion and improve hyperglycemia via the cAMP/PKA pathway in streptozocin-induced diabetic rats [103]. It is also able to inhibit PTGS2 and iNOS activity in BV2 microglia cells [104]. It can ameliorate high-glucose-mediated endothelial dysfunction by activating PKCBII and attenuating the expression of IL-1β, IKKB and NF-κB [105]. Furthermore, it has been shown to inhibit DPP-4 activity in vitro [106].

Caffeic acid and rosmarinic acid (an ester of caffeic acid and 3-(3,4-dihydroxyphenyl) lactic acid) are two phenolic compounds that have been widely studied for their health benefits. In vivo studies have reported that caffeic acid improves glucose homeostasis by modulating gluconeogenesis and glycogenesis (increasing glycogen synthase) via the cAMP/PKA signaling pathway and possibly CREB-1 [107]. Caffeic acid has also been demonstrated to inhibit AMY1C [108] and DPP-4 activity [109]. Some of its derivatives have been shown to improve insulin signaling and glucose uptake in 3T3LI adipocytes [110] as well as reduce oxidative stress, enhance PKA/CREB1 signaling and have antioxidant activity [111]. Caffeic acid phenethyl ester (CAPE) has been reported to improve metabolic syndrome, inhibit basal lipolysis and activate adipose tissue remodeling via the PPARγ signaling pathway in high-fat diet-induced obese rats [112]. It also has anti-inflammatory activity via suppression of NF-κB (p65) activation [113] and inhibition of PGTS2 [114,115].

Rosmarinic acid has previously been demonstrated to exert anti-inflammatory and antiglycative activity in diabetic mice. It significantly increased plasma insulin levels and reduced plasma glucose, liver IL-6, TNF-alpha, PGE2, and AGEs levels as well as the activity of PGTS2 and glyoxalase (GLO-1) [116]. Rosmarinic acid has been demonstrated to protect against diabetic nephropathy by inhibiting AGE formation and downregulating the expression of RAGE and PTGS2 [117,118]. It has also been reported to ameliorate hyperglycemia and insulin resistance and increase the expression of GLUT4 in high-fat diet-induced diabetic rats [119]. It can reverse palmitate-induced insulin resistance in muscle cells [120]. It has been shown to protect cardiomyocytes against inflammation and apoptosis in myocardial injury via increasing PPARγ and inhibiting PTGS2 activity [121]. It can also inhibit DPP-4 [122], PTP1B [123] and AMY1C [124,125] activity in vitro.

Oleanolic acid has been reported to enhance insulin secretion [126], likely implicating adherens junctions and the cAMP signaling pathway, as well as the modulation of PPARγ expression [127,128]. It has been linked with reduced inflammation via the inhibition of NF-κB activation in diabetic rat models [129]. In addition, oleanolic acid has GSK3B [130,131] and PTP1B [132] in vitro inhibitory activity.

Salvianolic acid B is polyphenol with diverse health benefits, including antioxidant and antidiabetic effects [133]. It has been reported to improve insulin resistance in experimental DM models [134], improve mitochondrial function, improve obesity-induced glucose tolerance via the PPAR pathway in high-fat diet-induced obese rats and in 3T3-L1 pre-adipocytes [135,136]. Its anti-inflammatory activity has been demonstrated in LPS-activated human aortic smooth muscle via inhibition of PTGS2 [137], inhibition of PTP1B [138] and of GSK3B activity [139] in vitro, as well as inhibition of NF-κB, iNOS and PGTS2 activity in high-fat diet-induced animal models of inflammation [140].

Molecular docking is commonly used to predict the interactions of molecules with biological targets in the context of drug discovery [141,142]. In this study, it was used to assess the nature of the molecular interactions between caffeic acid, apigenin, hispidulin, oleanolic acid, rosmarinic acid, salvianolic acid B and seven predicted core targets associated with the pathogenesis of T2DM. Rosmarinic acid showed the best SILE value amongst all ligands towards PTGS2, DPP4 and AMY1A. Salvianolic acid B showed the best SILE value towards PPARG, GSK3B and RELA. Caffeic acid showed the best SILE value towards PTB1B. All ligands showed various interactions with the amino acid residues of the protein targets, including hydrogen bonding interactions with crucial amino acid residues similar to those observed for the respective controls. These included interactions with Asp197, Asp300, and Glu233 for the AMY1A complexes [143,144], with Asp48, Ala217, and Ser216 for the PTP1B complexes [145,146], with Ser630, Tyr662, and Tyr631 for the DPP4 complexes [147,148], with Val135 for the GSK3B complexes [149,150], with Ser289 and Tyr473 for the PPARy complexes [151,152], with Ser530, Tyr355, and Tyr385 for the PTGS2 complexes [153,154] and lastly, with Ser45, Arg35, and Arg33 for the RELA complexes [155,156].

## 4. Conclusions

The antidiabetic effect of *S. officinalis* is predicted to involve a complex interplay between multiple compounds present in the aerial parts of this plant and multiple targets and pathways involved in the pathogenesis of T2DM. The key compounds target multiple core proteins and modulate various pathways including metabolic pathways, IL-17, NF-κB, TNF, cAMP, insulin resistance, and AGE-RAGE signaling. The identified *S. officinalis* compounds are likely to improve insulin secretion, insulin sensitivity and glucose tolerance and reduce inflammation, oxidative stress and AGEs formation thus mitigating the onset and progression of DM and its complications. Interestingly, some of the core targets/pathways identified in this study included some involved in the pathogenesis of cancer. Overall, this study enhances our understanding of the antidiabetic activity of *S. officinalis* and provides further insights for future drug discovery purposes.

## 5. Materials and Methods

### 5.1. Compounds Mining

The keywords “(“Salvia officinalis” OR “S. officinalis”) AND “NMR””, as well as “(“Salvia officinalis” OR “S. officinalis”) AND “Nuclear Magnetic Resonance””, were employed to query the SciFinder database (https://scifinder.cas.org) (accessed on 24 August 2023). This resulted in the retrieval of 68 research articles. Only studies (*n* = 22) reporting on purified compounds from the aerial parts *S. officinalis* unambiguously characterized by NMR were selected for further analysis. These were retrieved by activating the “substance” option on Scifinder when viewing the entry for a particular study. The canonical SMILES of the selected compounds were obtained from the PubChem (https://pubchem.ncbi.nlm.nih.gov/) (accessed on 9 October 2023) or Chemspider (http://www.chemspider.com) (accessed on 18 September 2023) databases. For compounds not found in these databases, ACD/ChemSketch (v. 12.0) was used to draw the structures and generate canonical SMILES. An Excel database file was created to store all compounds along with their canonical SMILES. The SMILES generated for each compound were analyzed using the SwissADME server to eliminate any duplicate structures.

### 5.2. Ranking of Mined Compounds

The freely accessible SwissADME tool (http://www.swissadme.ch) (accessed on 1 October 2023) was queried with the canonical SMILES of the selected phytochemicals to compute their physicochemical (molecular weight, lipophilicity, water solubility, rotatable bonds, H-bond donors/acceptors), pharmacokinetic (GI absorption, blood–brain barrier penetration, P-glycoprotein substrate/inhibition), and drug-likeness properties (by filtering for violations of Lipinski/Veber/Egan/Ghose/Muegge rules and assessing the Abbott bioavailability score) [157].

The canonical SMILES were also used to predict a drug-likeness score for each phytochemical using the freely accessible Molsoft L.L.C online tool (https://molsoft.com/mprop/) (accessed on 8 October 2023).

The chemical complexity (Cm) of each compound was obtained from PubChem.

Overall, only compounds with an oral Abbott bioavailability score ≥ 0.5 [158] and those that had previously demonstrated antidiabetic activity were selected for further analysis.

### 5.3. Identification of Compounds- and Disease-Associated Targets

Three online tools, namely, Similarity Ensemble Approach (SEA) (https://sea.bkslab.org/) (accessed on 17 October 2023), Swiss Target Prediction (STP) (http://www.swisstargetprediction.ch/) (accessed on 15 October 2023), and BindingDB (https://www.bindingdb.org/) (accessed on 19 October 2023) were queried with the canonical SMILES of the selected phytochemicals in order to predict the most probable protein/gene targets involved. To ensure a high confidence prediction, only targets in the highest-ranking category (i.e., with a score ≥ 0.70) were selected [159,160,161,162,163]. The annotations used for the selected gene/protein targets were standardized according to the UniProtKB database (https://www.uniprot.org/) (accessed on 22 October 2023). The targets were combined and duplicates were eliminated to retain a unique set of compound-associated targets.

For disease-associated targets related to T2DM, the following databases were searched: GeneCards (https://www.genecards.org/) (accessed on 23 October 2023), TTD (https://idrblab.net/ttd/) (accessed on 24 October 2023), DisGeNET (https://www.disgenet.org/) (accessed on 23 October 2023), KEGG (https://www.genome.jp/entry/H00409) (accessed on 23 October 2023), and MalaCards (https://www.malacards.org/) (accessed on 24 October 2023). The search terms used were “non-insulin dependent diabetes mellitus” (for DisGeNET only) and “type-2 diabetes mellitus” (for all others). The two sets of targets obtained (compound-associated and disease-associated) were combined to yield a subset of common targets using the VENNY 2.1 online tool (https://bioinfogp.cnb.csic.es/tools/venny/) (accessed on 25 October 2023). These common targets were used subsequently for network construction.

### 5.4. Network Construction and Analysis

Cytoscape v.3.9.1 was employed to construct the distinct networks, including the Protein–Protein Interaction (PPI), Compound-Target (CT), and Target-Pathways (TP) networks. The networks were constructed using a previously published methodology, with slight modifications [158].

#### 5.4.1. Construction of Protein–Protein Interaction (PPI) Network

The subset of common targets identified in Section 5.3 was uploaded onto the STRING online tool (https://string-db.org/) (accessed on 25 October 2023) to predict the PPI network involving most of our common targets. The analysis parameters were set with a minimum required interaction confidence score of 0.7, and the organism was specified as *Homo sapiens*. This meant that only human proteins with high interaction scores (≥0.7) were included in the network. The generated PPI network was exported to Cytoscape for further analysis and visualization with nodes representing proteins/genes, and edges representing interactions between them. The relevant topological parameters, such as degree centrality, betweenness centrality, and closeness centrality, were computed using the “Analyse Network” option in Cytoscape. To enhance visualization, the yFiles organic layout was applied. Additionally, the CytoHubba plugin was downloaded and installed in Cytoscape to identify and rank the core/hub targets within the PPI network.

#### 5.4.2. Construction of Compound-Target (CT) Network

The identified compounds and their associated common targets were systematically organized into a dataset on an Excel spreadsheet, formatted for compatibility with Cytoscape. The compounds were designated as source nodes in the first column, and the proteins/genes were assigned as target nodes in the second column. The remaining two columns contained the respective node attributes, facilitating the selective formatting of either class of nodes. The Excel spreadsheet file was imported into Cytoscape to generate the compound-target network. The network was further analyzed in Cytoscape for relevant topological parameters (centrality measures) using the “Analyze Network” function as well as the CytoHubba plugin as aforementioned to rank the core compounds linked with the targets within the PPI network. The yFiles organic layout was also applied to enhance visualization.

#### 5.4.3. Construction of Target-Pathways (TP) Network

The Database for Annotation, Visualization and Integrated Discovery (DAVID; https://david.ncifcrf.gov/tools.jsp) (accessed on 29 October 2023) was used to identify the key pathways linked to the subset of common targets (proteins) identified in Section 5.3. The latter was uploaded onto the left panel box labeled “Enter Gene List”. The “Official Gene Symbol” option was selected for “Select Identifiers”, and “Gene List” was chosen for the “List Type” option, and *Homo sapiens* was specified under “Select Species”. The submitted query generated an annotation summary results page, from which the “KEGG Pathway” file was downloaded as an Excel file containing the pathways, the enriched proteins/genes, and the statistical parameters of the enrichment analysis. The data were subsequently organized in an Excel spreadsheet as described in Section 5.4.2. The proteins were represented as source nodes in the first column, and the pathways were designated as target nodes in the second column. The remaining two columns contained the respective node attributes. The resulting file was uploaded into Cytoscape to construct the target-pathway (TP) network and visualize the connections between targets and associated pathways. The yFiles organic layout was applied again to enhance visualization.

Only pathways with a *p*-value ≤ 0.05, FDR ≤ 0.05, and a Benjamini value of ≤0.05 were selected [158,163] and terms not related to diabetes mellitus were removed.

### 5.5. GO and KEGG Pathway Enrichment Analysis

The cellular component, biological process, molecular function, and KEGG pathways associated with the common targets were identified using the ShinyGO program v. 0.77 (http://bioinformatics.sdstate.edu/go/) (accessed on 29 October 2023). The standardized gene symbols of each target were uploaded to ShinyGO, specifying *Homo sapiens* as the species and setting the false discovery rate (FDR) cut-off value at 0.05 [164]. The results were presented as lollipop plots.

### 5.6. Molecular Docking: Preparation and Simulation

#### 5.6.1. Protein Target Preparation

The protocol used for protein target preparation was based on a previous method with slight modifications [165]. The X-ray crystallography structural data of selected key targets: AMY1A (2QV4), NF-κB (p65 subunit) (1NFI), PTP1B (4Y14), DPP4 (5T4B), GSK3B (4AFJ), PPARG (5Y2T) and PTGS2 (5IKQ) were downloaded in .pdb format with their co-crystalized ligand (serving as controls) from the RCSB PDB database (http://www.rcsb.org/) (assessed on 30 November 2023). Only proteins from *Homo sapiens* with refinement resolutions between 1.7 and 2.0 Å were selected, with the exception of 5IKQ and 1NFI, which had resolutions of 2.14 Å and 2.70 Å, respectively.

The protein targets were prepared using the tools and protocols available in MOE 2015. The preparatory process included the removal of water molecules and other co-crystalized molecules, followed by protonation, partial charges and energy minimization using the QuickPrep function in MOE. The fully prepared and optimized 3D structures were saved in .moe format for docking.

#### 5.6.2. Binding/Docking Site Prediction

The docking sites were identified as the binding sites of the respective co-crystalized ligands for all target proteins except for NF-κB, for which the docking site was the DNA binding site on the protein subunits [156].

#### 5.6.3. Validation of Docking

A dummy docking was carried out using the X-ray structures of the target proteins and their co-crystallized ligands. Each isolated co-crystallized ligand was re-docked onto the binding site of its corresponding target protein. This was repeated several times with a different scoring function—ASE, Affinity dG, Alpha HB, Electron Density, GBVI/WSA dG and London dG/- each time. The docked binding pose for each scoring function was compared to the experimentally determined pose in the X-ray complex. A RMSD value ≤ 2.0 Å (relative to the native binding pose of the control ligand) was considered a good validation of the docking results [166]. As the scoring functions produced a docking pose within 2.0 Å of the experimental pose, the default scoring function of London dG/GBVI/WSA dG with the induced fit refinement method was adopted for the study.

#### 5.6.4. Docking Simulations

Docking simulations were performed on an Intel core i7 CPU @ 2.00 GHz, 2.60 GHz using MOE and following a previously reported protocol with slight modifications [167]. All ligands were docked using the Triangular Matcher/Rigid Receptor (default) method. The poses generated were scored using the London dG function and subsequently re-scored using the GBVI/WSA dG scoring function [165]. The protein-ligand docking poses and their respective scores were saved in a database in .mdb format and the (2D and 3D) interactions with the target proteins were visualized using Drug Discovery Studio v. 16.0 (Dassault Systèmes, Paris, France). The docking scores of the top-ranking poses for each selected ligand were normalized by computing the corresponding size-independent ligand efficiency (SILE) values using Equation (1) below [168].

(1) $$SILE\;value=-(\frac{D}{N^{0.3}})$$

where D is the docking score and N is the number of heavy atoms in the ligand.

## Figures and Tables

**Table 1:** Docking scores (in kcal/mol), SILE values and interacting amino acid residues for the ligands docked with prostaglandin G/H synthase 2 (PTGS2).

Ligand	Docking Score	SILE Value	Interacting Amino Acid Residues
Caffeic acid	−5.1	2.37	(Leu352, Tyr312) ^a^, Arg550 ^d^, (Leu352, Val523) ^c^
Apigenin	−6.4	2.62	(His90, Tyr355, Tyr385) ^a^, (Val523, Ala527, Leu352,Val349, Ser353) ^c^
Hispidulin	−6.5	2.58	Ser530 ^a^, (Leu352, Ala527, Val523, Val349, Gly526) ^c^
Oleanolic acid	8.3	−2.91	Tyr385 ^b^
Rosmarinic acid	−7.7	2.90	(Leu352, Tyr355, Arg120) ^a^, Arg120 ^d^, (Ser530, Tyr385) ^b^, (Val523, Leu352, Gly526, Ala527) ^c^
Salvianolic acid B	−2.3	0.71	(Ser119, Tyr355) ^a^, (Arg513, Arg120) ^d^, (Val523, Pro86) ^c^, Glu524 ^e^
PubChem CID: 4037 (Control)	−7.0	2.90	(Ser530, Tyr385) ^a^, (Val116, Leu531, Ala527) ^f^, (Ala527, Val523, Val349) ^c^

a—conventional hydrogen bond, b—carbon-hydrogen bond, c—Pi interactions, d—salt bridge/attractive charges, e—Pi-ions, f—Alkyl.

**Table 2:** Docking scores (in kcal/mol), SILE values and interacting amino acid residues for the ligands docked with peroxisome proliferator-activated receptor gamma (PPARγ).

Ligand	Docking Score	SILE Value	Interacting Amino Acid Residues
Caffeic acid	−5.0	2.33	Ser289 ^a^, (Met364, Arg288, Leu330, Cys285) ^c^
Apigenin	−6.0	2.46	(Ser342, Cys285) ^a^, Arg288 ^b^, (Arg288, Leu330, Cys285, Met364, Ile341, Gly284) ^c^
Hispidulin	−6.6	2.60	(Ser289, Tyr473) ^a^, (Tyr473, His449 His323) ^b^, (Arg288, His449, Met364,Leu330, Cys285) ^c^
Oleanolic acid	−5.6	1.95	Ile341 ^e^, (Phe287, Arg280) ^c^
Rosmarinic acid	−7.3	2.76	(Ser342, Tyr327) ^a^, (Cys285, Met364, Gly284 and Tyr327) ^c^
Salvianolic acid B	−9.5	2.91	(Ser289, Ser342, Glu343, Glu291, Cys285) ^a^, Cys285 ^d^, (Arg288, Leu330, Gly291, Arg 280) ^c^
PubChem CID: 76965111 (Control)	−9.7	3.36	(Ser289 Tyr473) ^a^, (Cys285, Leu255) ^e^, (Leu353, Leu330, Met348, Val339) ^c^

a—conventional hydrogen bond, b—carbon-hydrogen bond, c—Pi interactions, d—Pi-ions, e—Alkyl.

**Table 3:** Docking scores (in kcal/mol), SILE values and interacting amino acid residues for the ligands docked with protein tyrosine phosphatase 1B (PTP1B).

Ligand	Docking Score	SILE Value	Interacting Amino Acid Residues
Caffeic acid	−6.0	2.78	(Asp48, Ala217, Ser216, Arg221) ^a^, Arg221 ^d^
Apigenin	−5.0	2.03	Asp48 ^e^, (Tyr46, Ala217) ^c^
Hispidulin	−5.4	2.15	Ser216 ^b^, Arg221 ^d^, (Ala217, Tyr46) ^c^
Oleanolic acid	−4.8	1.67	Asp48 ^a^, Lys120 ^d^, Lys116 ^c^, (Ala217, Tyr46, Phe182) ^f^
Rosmarinic acid	−5.9	2.20	(Arg221, Tyr46) ^a^, lys120 ^d^, (Ala217, Phe182, Arg47) ^c^
Salvianolic acid B	−6.9	2.12	(Asp48, Gln262, Tyr46, Ser118, Leu119) ^a^, Asp181 ^b^, (Lys116, Phe182, Ala217) ^c^, Lys120 ^e^, Lys120 ^d^
PubChem CID: 91826021(Control)	−8.7	3.32	(Asp48, Phe182, Ala217, Ser216, Gly220, Ile219) ^a^, (Phe182, Ala217) ^c^, (Arg221, Asp181) ^d^

a—conventional hydrogen bond, b—carbon-hydrogen bond, c—Pi interactions, d—salt bridge/attractive charges, e—Pi-ions, f—Alkyl.

**Table 4:** Docking scores (in kcal/mol), SILE values and interacting amino acid residues for the ligands docked with dipeptidyl peptidase 4 (DPP 4).

Ligand	Docking Score	SILE Value	Interacting Amino Acid Residues
Caffeic acid	−4.9	2.28	(Ser630, Tyr631, Trp629) ^a^, Tyr547 ^c^, Lys554 ^d^
Apigenin	−5.1	2.09	Glu205, Arg125, Lys554) ^a^ Tyr547 ^c^
Hispidulin	−5.9	2.32	Asp545 ^a^, (Trp666, Tyr547) ^c^ Arg125 ^d^, Ser630 ^b^, Glu205 ^b^, Tyr662 ^b^
Oleanolic acid	−6.1	2.15	Arg125 ^d^, Tyr752 ^b^, (Trp629, Tyr547) ^f^, Tyr547 ^c^
Rosmarinic acid	−6.8	2.57	(Asp709, Lys122) ^a^, (Ser630, Tyr547) ^b^, Trp629 ^e^, (Arg125, Tyr547) ^c^
Salvianolic acid B	−8.0	2.45	(Tyr752 ^b^, Tyr48) ^a^, (Asn562, Trp629) ^b^, (Arg125, Lys554) ^d^, (Tyr547, Trp629) ^c^
PubChem CID: 137348565 (Control)	−8.3	2.84	(Tyr662, Tyr631) ^a^, Ser630 ^b^, (Val656, Val711) ^f^, (Asp663, Glu206, Glu205) ^d^, Trp629 ^c^, Phe357 ^c^

a—conventional hydrogen bond, b—carbon-hydrogen bond, c—Pi interactions, d—salt bridge/attractive charges, e—Pi-ions, f—Alkyl.

**Table 5:** Docking scores (in kcal/mol), SILE values and interacting amino acid residues for the ligands docked with glycogen synthase kinase-3 beta (GSK3B).

Ligand	Docking Score	SILE Value	Interacting Amino Acid Residues
Caffeic acid	−5.1	2.35	Val135 ^a^, Lys85 ^d^, (Leu188, Ala83) ^c^
Apigenin	−5.7	2.33	(Glu97, Val135) ^a^, Cys199 ^b^, (Ala83, Lys85, Val110, leu188, Leu132) ^c^
Hispidulin	−6.1	2.40	(Glu97, Val135) ^a^, (Cys199, Val135) ^b^, (Ala83, Lys85, Val110, Leu188, Leu132, Met101) ^c^, Asp200 ^g^
Oleanolic acid	−6.1	2.15	(Asn186, Tyr 134) ^a^, Lys60 ^d^, (Arg141, Cys199, Phe67) ^f^
Rosmarinic acid	−6.3	2.37	(Asp133, Val135) ^a^, (Ala83, Lys199, Leu188, Val70) ^c^, Lys85 ^d^
Salvianolic acid B	−8.4	2.58	(Asp181, Lys183, Thr138, Asn64, Arg141) ^a^, (Gly63, Lys85) ^b^ (Phe67, Lys85, Val70, Cys199) ^c^, Arg141 ^e^
PubChem CID: 56643097 (Control)	−7.0	2.73	Val135 ^a^, (Ile62, Ala83, Val70, Lys85, Leu158, Phe67) ^c^, Lys85 ^f^

a—conventional hydrogen bond, b—carbon-hydrogen bond, c—Pi interactions, d—salt bridge/attractive charges, e—Pi-ions, f—Alkyl, g—pi-donor hydrogen bond.

**Table 6:** Docking scores (in kcal/mol), SILE values and interacting amino acid residues for the ligands docked with amylase-alpha 1A (AMY1A).

Ligand	Docking Score	SILE Value	Interacting Amino Acid Residues
Caffeic acid	−4.8	2.24	(Asp197, Gln63) ^a^, Trp59 ^e^, Tyr62 ^c^
Apigenin	−5.7	2.31	(Asp197, Gln63) ^a^, (Trp59, Tyr62) ^c^
Hispidulin	−5.9	2.32	(Asp197 ^a^, Gln63) ^a^, Trp59 ^e^, (Trp59, Tyr62) ^c^
Oleanolic acid	−6.4	2.25	Asp197 ^a^, (Trp59, His305, Tyr62, Leu162) ^c^, (Trp59, His305, Leu162, His 101, Ala198) ^f^
Rosmarinic acid	−6.5	2.44	(His201, Thr163, Arg 195 a) ^a^, Asp197 ^b^, (Arg195, Tyr62) ^d^, (Ala198, Leu165, leu162) ^c^
Salvianolic acid B	−7.9	2.42	Gly304 ^a^, Asp197 ^a^, Lys200 ^b^, Lys200 ^e^
PubChem CID: 24755467 (Control)	−9.4	2.83	(Gly164, Gln63, Asp300, Trp69, Glu233, Gln63, His101, Arg195, His299, Tyr62, Asn105, Ala106, Val107, Leu162) ^a^, (Asp300, Glu233, Asp197) ^d^

a—conventional hydrogen bond, b—carbon-hydrogen bond, c—Pi interactions, d—salt bridge/attractive charges, e—Pi-ions, f—Alkyl.

**Table 7:** Docking scores (in kcal/mol), SILE values and interacting amino acid residues for the ligands docked with transcription factor p65 NF-κB (RELA).

Ligand	Docking Score	SILE Value	Interacting Amino Acid Residues
Caffeic acid	−5.0	2.33	(Arg41, Ile118) ^a^, (Arg35, Ala43, Val91) ^c^
Apigenin	−5.3	2.15	(Ser42, Val91) ^c^
Hispidulin	−5.3	2.10	Tyr36 ^a^, Ser42 ^b^, (Ala43, Arg33) ^c^, Val91 ^c^
Oleanolic acid	−5.1	1.80	Gly44 ^a^, Ala43 ^f^
Rosmarinic acid	−6.1	2.28	(Ser44, Glu39, Gly92, Glu89, Gln119) ^a^, Val91 ^c^
Salvianolic acid B	−8.0	2.43	(Ser45, Arg33) ^a^, Lys56 ^e^, (Arg33, Arg35) ^d^, (Gly44, Arg 33) ^b^, (Ala43, Lys56, Arg35, Met32) ^c^
PubChem CID: 9881652 (Control)	−5.2	2.13	(Ser51, Lys56, Arg35, Ser45) ^a^, Met32 ^c^

a—conventional hydrogen bond, b—carbon-hydrogen bond, c—Pi interactions, d—salt bridge/attractive charges, e—Pi-ions, f—Alkyl.

**Table 8:** Molecular interactions between the ligands with the highest SILE values and each target protein.

Target Protein	Ligand	Docking Score (kcal/mol)	SILE Value	Interacting Residues	Distance (Å)	Category	Type
AMY1A (2QV4)	Rosmarinic acid	−6.479	2.44	Thr163	3.131	H-Bond	Conventional H-Bond
				Arg195	3.219	H-Bond	Conventional H-Bond
				His201	2.010	H-Bond	Conventional H-Bond
				Asp197	2.564	H-Bond	Carbon H-Bond
				Leu162	3.910	Hydrophobic	Pi-Sigma
				Ala198	5.492	Hydrophobic	Pi-Alkyl
				Leu165	5.162	Hydrophobic	Pi-Alkyl
				Arg195	5.297	Electrostatic	Attractive Charge
				Tyr62	3.314	Electrostatic	Pi-Anion
DPP4 (5T4B)	Rosmarinic acid	−6.834	2.57	Lys122	3.134	H-Bond	Conventional H-Bond
				Asp709	2.190	H-Bond	Conventional H-Bond
				Asp709	2.537	H-Bond	Conventional H-Bond
				Ser630	3.712	H-Bond	Carbon H-Bond
				Tyr547	2.944	H-Bond	Pi-Donor H-Bond
				Trp629	4.749	Electrostatic	Pi-Anion
				Trp629	3.626	Electrostatic	Pi-Anion
				Trp629	3.351	Electrostatic	Pi-Anion
				Tyr547	5.643	Hydrophobic	Pi-Pi T-shaped
				Arg125	4.342	Hydrophobic	Pi-Alkyl
PTGS2 (5IKQ)	Rosmarinic Acid	−7.714	2.90	Tyr355	2.399	H-Bond	
				Tyr385	2.266	H-Bond	
				Arg120	2.524	H-Bond	H-Bond
				Leu352	2.709	H-Bond	H-Bond
				Ser530	2.700	H-Bond	H-Bond
				Tyr385	2.801	H-Bond	Pi-Donor H-Bond
				Gly526	3.766	Hydrophobic	Amide-Pi Stacked
				Leu352	5.451	Hydrophobic	Pi-Alkyl
				Val523	3.778	Hydrophobic	Pi-Alkyl
				Ala527	4.950	Hydrophobic	Pi-Alkyl
				Arg120	2.245	H-Bond;Electrostatic	Salt Bridge;Attractive Charge
				Arg120	5.054	Electrostatic	Attractive Charge
PTP1B (4Y14)	Caffeic acid	−6.010	2.78	Arg221	2.399	H-Bond;Electrostatic	Salt Bridge
				Ser216	2.266	H-Bond	Conventional H-Bond
				Ala217	2.524	H-Bond	Conventional H-Bond
				Arg221	2.709	H-Bond	Conventional H-Bond
				Asp48	2.700	H-Bond	Conventional H-Bond
				Asp48	2.801	H-Bond	Conventional H-Bond
				Cys215	3.766	H-Bond	
				Gly220	5.451	H-Bond	
				Ala217	3.778	Hydrophobic	Pi-Sigma
GSK3B (4AFJ)	Salvianolic acid B	−8.447	2.58	Asn64	3.083	H-Bond	Conventional H-Bond
				Asn64	3.099	H-Bond	
				Arg141	3.071	H-Bond	Conventional H-Bond
				Lys183	2.922	H-Bond	Conventional H-Bond
				Asp181	2.613	H-Bond	Conventional H-Bond
				Thr138	2.808	H-Bond	Conventional H-Bond
				Lys85	3.039	H-Bond	
				GLY63	3.702	H-Bond	Carbon H-Bond
				ARG141	3.966	Electrostatic	Pi-Cation
				VAL70	3.909	Hydrophobic	Pi-Sigma
				PHE67	4.971	Hydrophobic	Pi-Pi T-shaped
				LYS85	5.156	Hydrophobic	Pi-Alkyl
NF-κB (1NFI)	Salvianolic acid B	−7.963	2.43	ARG33	2.838	H-Bond	Conventional H-Bond
				SER45	3.312	H-Bond	Conventional H-Bond
				ARG33	1.790	H-Bond	Conventional H-Bond
				ARG33	3.767	H-Bond	Carbon H-Bond
				GLY44	3.499	H-Bond	Carbon H-Bond
				LYS56	4.170	H-Bond;Electrostatic	Pi-Cation;Pi-Donor H-Bond
				LYS56	3.485	H-Bond;Electrostatic	Pi-Cation;Pi-Donor H-Bond
				ARG35	3.123	H-Bond;Electrostatic	Salt Bridge
				ARG35	2.899	H-Bond;Electrostatic	Salt Bridge
				ARG35	2.899	H-Bond;Electrostatic	Salt Bridge
				ARG33	3.177	Electrostatic	Attractive Charge
				ARG35	5.023	Electrostatic	Attractive Charge
				MET32	3.348	Hydrophobic	Pi-Sigma
				LYS56	5.233	Hydrophobic	Pi-Alkyl
				ARG35	5.003	Hydrophobic	Pi-Alkyl
				ALA43	4.395	Hydrophobic	Pi-Alkyl
PPARγ (5Y2T)	Salvianolic acid B	−9.519	2.91	SER289	3.169	H-Bond	
				SER342	3.209	H-Bond	
				SER289	2.315	H-Bond	
				CYS285	3.054	H-Bond	Conventional H-Bond
				CYS285	3.124	H-Bond	Conventional H-Bond
				SER342	3.088	H-Bond	Conventional H-Bond
				GLU343	2.134	H-Bond	Conventional H-Bond
				GLU291	2.502	H-Bond	Conventional H-Bond
				SER289	2.315	H-Bond	Conventional H-Bond
				CYS285	3.709	H-Bond	Pi-Donor H-Bond
				ARG280	4.644	Hydrophobic	Amide-Pi Stacked
				ARG280	5.406	Hydrophobic	Pi-Alkyl
				ARG288	4.900	Hydrophobic	Pi-Alkyl
				LEU330	4.615	Hydrophobic	Pi-Alkyl
				GLU291	3.360	Electrostatic	Pi-Anion

## Data Availability

The raw data supporting the conclusions of this article will be made available by the authors on request.

## Supplementary Materials

The following supporting information can be downloaded at: https://www.mdpi.com/article/10.3390/plants13202892/s1. Table S1: ADME/Drug-likeness predictions of *S. officinalis* phytochemicals (compounds selected for the network pharmacology study are in bold); Table S2: List of biological targets predicted to be associated with *S. officinalis* selected compounds; Table S3: List of biological targets predicted to be associated with both *S. officinalis* compounds and T2DM; Table S4: List of predicted T2DM targets associated with *S. officinalis* compounds, used to construct the compound-target (CT) network; Table S5: Topological parameters of the predicted common targets in the protein-protein interaction network.

## References

[1] Khan M.A.B., Hashim M.J., King J.K., Govender R.D., Mustafa H., Al Kaabi J. (2020). Epidemiology of Type 2 Diabetes—Global Burden of Disease and Forecasted Trends. J. Epidemiol. Glob. Health.

[2] Perreault L., Skyler J.S., Rosenstock J. (2021). Novel therapies with precision mechanisms for type 2 diabetes mellitus. Nat. Rev. Endocrinol..

[3] Singh S., Kriti M., Anamika K.S., Sarma D.K., Verma V., Nagpal R., Mohania D., Tiwari R., Kumar M. (2024). Deciphering the complex interplay of risk factors in type 2 diabetes mellitus: A comprehensive review. Metabol. Open.

[4] Saeedi P., Petersohn I., Salpea P., Malanda B., Karuranga S., Unwin N., Colagiuri S., Guariguata L., Motala A.A., Ogurtsova K. (2019). Global and regional diabetes prevalence estimates for 2019 and projections for 2030 and 2045: Results from the International Diabetes Federation Diabetes Atlas, 9th edition. Diabetes Res. Clin. Pract..

[5] Sun H., Saeedi P., Karuranga S., Pinkepank M., Ogurtsova K., Duncan B.B., Stein C., Basit A., Chan J.C.N., Mbanya J.C. (2022). IDF Diabetes Atlas: Global, regional and country-level diabetes prevalence estimates for 2021 and projections for 2045. Diabetes Res. Clin. Pract..

[6] DeFronzo R.A., Ferrannini E., Groop L., Henry R.R., Herman W.H., Holst J.J., Hu F.B., Kahn C.R., Raz I., Shulman G.I. (2015). Type 2 diabetes mellitus. Nat. Rev. Dis. Primers.

[7] Li Y., Liu Y., Liu S., Gao M., Wang W., Chen K., Huang L., Liu Y. (2023). Diabetic vascular diseases: Molecular mechanisms and therapeutic strategies. Signal Transduct. Target. Ther..

[8] Ansari P., Samia J.F., Khan J.T., Rafi M.R., Rahman M.S., Rahman A.B., Abdel-Wahab Y.H.A., Seidel V. (2023). Protective Effects of Medicinal Plant-Based Foods Against Diabetes: A Review on Pharmacology, Phytochemistry, and Molecular Mechanisms. Nutrients.

[9] Furman B.L., Candasamy M., Bhattamisra S.K., Veettil S.K. (2020). Reduction of blood glucose by plant extracts and their use in the treatment of diabetes mellitus; discrepancies in effectiveness between animal and human studies. J. Ethnopharmacol..

[10] Alam S., Dhar A., Hasan M., Richi F.T., Emon N.U., Aziz M.A., Mamun A.A., Chowdhury M.N.R., Hossain M.J., Kim J.K. (2022). Antidiabetic Potential of Commonly Available Fruit Plants in Bangladesh: Updates on Prospective Phytochemicals and Their Reported MoAs. Molecules.

[11] Ansari P., Akther S., Hannan J.M.A., Seidel V., Nujat N.J., Abdel-Wahab Y.H.A. (2022). Pharmacologically Active Phytomolecules Isolated from Traditional Antidiabetic Plants and Their Therapeutic Role for the Management of Diabetes Mellitus. Molecules.

[12] Ansari P., Choudhury S.T., Seidel V., Rahman A.B., Aziz M.A., Richi A.E., Rahman A., Jafrin U.H., Hannan J.M.A., Abdel-Wahab Y.H.A. (2022). Therapeutic Potential of Quercetin in the Management of Type-2 Diabetes Mellitus. Life.

[13] Asante D.B., Wiafe G.A. (2023). Therapeutic Benefit of Vernonia amygdalina in the Treatment of Diabetes and Its Associated Complications in Preclinical Studies. J. Diabetes Res..

[14] Chhabria S., Mathur S., Vadakan S., Sahoo D.K., Mishra P., Paital B. (2022). A Review on Phytochemical and Pharmacological Facets of Tropical Ethnomedicinal Plants as Reformed DPP-IV Inhibitors to Regulate Incretin Activity. Front. Endocrinol..

[15] Roy S., Ghosh A., Majie A., Karmakar V., Das S., Dinda S.C., Bose A., Gorain B. (2024). Terpenoids as potential phytoconstituent in the treatment of diabetes: From preclinical to clinical advancement. Phytomedicine.

[16] Raal A., Orav A., Arak E. (2007). Composition of the essential oil of *Salvia officinalis* L. from various European countries. Nat. Prod. Res..

[17] Delamare A.P.L., Moschen-Pistorello I.T., Artico L., Atti-Serafini L., Echeverrigaray S. (2007). Antibacterial activity of the essential oils of *Salvia officinalis* L. and *Salvia triloba* L. cultivated in South Brazil. Food Chem..

[18] Ghorbani A., Esmaeilizadeh M. (2017). Pharmacological properties of Salvia officinalis and its components. J. Tradit. Complement. Med..

[19] Horváthová E., Srančíková A., Regendová-Sedláčková E., Melušová M., Meluš V., Netriová J., Krajčovičová Z., Slameňová D., Pastorek M., Kozics K. (2016). Enriching the drinking water of rats with extracts of Salvia officinalis and Thymus vulgaris increases their resistance to oxidative stress. Mutagenesis.

[20] Mansourabadi A.H., Sadeghi H.M., Razavi N., Rezvani E. (2016). Anti-inflammatory and analgesic properties of salvigenin, Salvia officinalis flavonoid extracted. Adv. Herb. Med..

[21] Bozin B., Mimica-Dukic N., Samojlik I., Jovin E. (2007). Antimicrobial and Antioxidant Properties of Rosemary and Sage (*Rosmarinus officinalis* L. and *Salvia officinalis* L., Lamiaceae) Essential Oils. J. Agric. Food Chem..

[22] Khedher M.R., Hammami M., Arch J.R.S., Hislop D.C., Eze D., Wargent E.T., Kępczyńska M.A., Zaibi M.S. (2018). Preventive Effects of Salvia officinalis Leaf Extract on Insulin Resistance and Inflammation in a Model of High Fat Diet-Induced Obesity in Mice that Responds to Rosiglitazone. PeerJ.

[23] Eidi M., Eidi A., Zamanizadeh H. (2005). Effect of *Salvia officinalis* L. leaves on serum glucose and insulin in healthy and streptozotocin-induced diabetic rats. J. Ethnopharmacol..

[24] Behradmanesh S., Derees F., Rafieian-Kopaei M. (2013). Effect of Salvia officinalis on Diabetic Patients. J. Renal Inj. Prev..

[25] Kianbakht S., Dabaghian F.H. (2013). Improved glycemic control and lipid profile in hyperlipidemic type 2 diabetic patients consuming *Salvia officinalis* L. leaf extract: A randomized placebo. Controlled clinical trial. Complement. Ther. Med..

[26] Hopkins A.L. (2007). Network pharmacology. Nat. Biotechnol..

[27] Zhou Z., Chen B., Chen S., Lin M., Chen Y., Jin S., Chen W., Zhang Y. (2020). Applications of Network Pharmacology in Traditional Chinese Medicine Research. Evid.-Based Complement. Alternat. Med..

[28] Li S., Fan T.P., Jia W., Lu A., Zhang W. (2014). Network pharmacology in traditional Chinese medicine. Evid.-Based Complement. Altern. Med..

[29] Li W., Yuan G., Pan Y., Wang C., Chen H. (2017). Network pharmacology studies on the bioactive compounds and action mechanisms of natural products for the treatment of diabetes mellitus: A review. Front. Pharmacol..

[30] An W., Huang Y., Chen S., Teng T., Shi Y., Sun Z., Xu Y. (2021). Mechanisms of Rhizoma Coptidis Against Type 2 Diabetes Mellitus Explored by Network Pharmacology Combined with Molecular Docking and Experimental Validation. Sci. Rep..

[31] Ge Q., Chen L., Tang M., Zhang S., Liu L., Gao L., Ma S., Kong M., Yao Q., Feng F. (2018). Analysis of mulberry leaf components in the treatment of diabetes using network pharmacology. Eur. J. Pharmacol..

[32] Pan L., Li Z., Wang Y., Zhang B., Liu G., Liu J. (2020). Network pharmacology and metabolomics study on the intervention of traditional Chinese medicine Huanglian Decoction in rats with type 2 diabetes mellitus. J. Ethnopharmacol..

[33] Wang N., Zhu F., Shen M., Qiu L., Tang M., Xia H., Chen L., Yuan Y., Ma S., Chen K. (2019). Network pharmacology-based analysis on bioactive anti-diabetic compounds in Potentilla discolor Bunge. J. Ethnopharmacol..

[34] Xu X., Niu L., Liu Y., Pang M., Lu W., Xia C., Wang Q. (2020). Study on the Mechanism of Gegen Qinlian Decoction for Treating Type II Diabetes Mellitus by Integrating Network Pharmacology and Pharmacological Evaluation. J. Ethnopharmacol..

[35] Zhu C., Cai T., Jin Y., Chen J., Liu G., Xu N., Shen R., Chen Y., Han L., Wang S. (2020). Artificial intelligence and network pharmacology based investigation of pharmacological mechanism and substance basis of Xiaokewan in treating diabetes. Pharmacol. Res..

[36] Fang J., Wang L., Wu T., Yang C., Gao L., Cai H., Liu J., Fang S., Chen Y., Tan W. (2017). Network pharmacology-based study on the mechanism of action for herbal medicines in Alzheimer treatment. J. Ethnopharmacol..

[37] Nazir S.S., Goel D., Vohora D. (2023). A network pharmacology based approach to decipher the pharmacological mechanisms of Salvia officinalis in neurodegenerative disorders. Res. Sq..

[38] Malik A., Morya R.K., Bhadada S.K., Rana S. (2018). Type 1 diabetes mellitus: Complex interplay of oxidative stress, cytokines, gastrointestinal motility and small intestinal bacterial overgrowth. Eur. J. Clin. Investig..

[39] Santiago J.A., Potashkin J.A. (2013). Shared dysregulated pathways lead to Parkinson’s disease and diabetes. Trends Mol. Med..

[40] Sanches J.M., Zhao L.N., Salehi A., Wollheim C.B., Kaldis P. (2023). Pathophysiology of type 2 diabetes and the impact of altered metabolic interorgan crosstalk. FEBS J..

[41] Ononamadu C.J., Alhassan A.J., Ibrahim A., Imam A.A., Ihegboro G.O., Owolarafe T.A., Sule M.S. (2019). Methanol-Extract/Fractions of Dacryodes edulis Leaves Ameliorate Hyperglycemia and Associated Oxidative Stress in Streptozotocin-Induced Diabetic Wistar Rats. J. Evid. Based Integr. Med..

[42] Zięba A., Stępnicki P., Matosiuk D., Kaczor A.A. (2022). What are the challenges with multi-targeted drug design for complex diseases?. Expert Opin. Drug Discov..

[43] Cabri W., Cantelmi P., Corbisiero D., Fantoni T., Ferrazzano L., Martelli G., Mattellone A., Tolomelli A. (2021). Therapeutic Peptides Targeting PPI in Clinical Development: Overview, Mechanism of Action and Perspectives. Front. Mol. Biosci..

[44] Rout T., Mohapatra A., Kar M., Patra S., Muduly D. (2024). Centrality Measures and Their Applications in Network Analysis: Unveiling Important Elements and Their Impact. Procedia Comput. Sci..

[45] Chaudhury A., Duvoor C., Reddy Dendi V.S., Kraleti S., Chada A., Ravilla R., Marco A., Shekhawat N.S., Montales M.T., Kuriakose K. (2017). Clinical Review of Antidiabetic Drugs: Implications for Type 2 Diabetes Mellitus Management. Front. Endocrinol..

[46] Nakao S., Ogtata Y., Shimizu E., Yamazaki M., Furuyama S., Sugiya H. (2002). Tumor necrosis factor alpha (TNF-alpha)-induced prostaglandin E2 release is mediated by the activation of cyclooxygenase-2 (COX-2) transcription via NFkappaB in human gingival fibroblasts. Mol. Cell. Biochem..

[47] Ke B., Zhao Z., Ye X., Gao Z., Manganiello V., Wu B., Ye J. (2015). Inactivation of NF-κB p65 (RelA) in Liver Improves Insulin Sensitivity and Inhibits cAMP/PKA Pathway. Diabetes.

[48] Wang X., Tao Y., Huang Y., Zhan K., Xue M., Wang Y., Ruan D., Liang Y., Huang X., Lin J. (2017). Catalase Ameliorates Diabetes-Induced Cardiac Injury through Reduced p65/RelA-Mediated Transcription of BECN1. J. Cell. Mol. Med..

[49] Zammit N.W., Wong Y.Y., Walters S.N., Warren J., Barry S.C., Grey S.T. (2023). RELA Governs a Network of Islet-Specific Metabolic Genes Necessary for Beta Cell Function. Diabetologia.

[50] Yan H., He L., Lv D., Yang J., Yuan Z. (2024). The Role of the Dysregulated JNK Signaling Pathway in the Pathogenesis of Human Diseases and Its Potential Therapeutic Strategies: A Comprehensive Review. Biomolecules.

[51] Yung J.H.M., Giacca A. (2020). Role of c-Jun N-Terminal Kinase (JNK) in Obesity and Type 2 Diabetes. Cells.

[52] Ijaz A., Tejada T., Catanuto P., Xia X., Elliot S.J., Lenz O., Jauregui A., Saenz M.O., Molano R.D., Pileggi A. (2009). Inhibition of C-jun N-terminal kinase improves insulin sensitivity but worsens albuminuria in experimental diabetes. Kidney Int..

[53] Martín-Vázquez E., Cobo-Vuilleumier N., López-Noriega L., Lorenzo P.I., Gauthier B.R. (2023). The PTGS2/COX2-PGE2 signaling cascade in inflammation: Pro or anti? A case study with type 1 diabetes mellitus. Int. J. Biol. Sci..

[54] Helmersson J., Vessby B., Larsson A., Basu S. (2004). Association of type 2 diabetes with cyclooxygenase-mediated inflammation and oxidative stress in an elderly population. Circulation.

[55] Shanmugam N., Todorov I.T., Nair I., Omori K., Reddy M.A., Natarajan R. (2006). Increased expression of cyclooxygenase-2 in human pancreatic islets treated with high glucose or ligands of the advanced glycation endproduct-specific receptor (AGER), and in islets from diabetic mice. Diabetologia.

[56] Tan G.S.Q., Morton J.I., Wood S., Trevaskis N.L., Magliano D.J., Windsor J., Shaw J.E., Ilomäki J. (2024). COX2 inhibitor use and type 2 diabetes treatment intensification: A registry-based cohort study. Diabetes Res. Clin. Pract..

[57] Bag S., Das S., Bagchi C., Tripathi S.K. (2014). Aspirin Potentiates Blood Glucose Lowering Effect of Glimepiride-Pioglitazone Combination in Streptozotocin-Induced Diabetic Rats. Indian J. Pharmacol..

[58] Herzig S., Long F., Jhala U.S., Hedrick S., Quinn R., Bauer A., Rudolph D., Schutz G., Yoon C., Puigserver P. (2001). CREB regulates hepatic gluconeogenesis through the coactivator PGC-1. Nature.

[59] Xu Y., Song R., Long W., Guo H., Shi W., Yuan S., Xu G., Zhang T. (2018). CREB1 Functional Polymorphisms Modulating Promoter Transcriptional Activity Are Associated with Type 2 Diabetes Mellitus Risk in Chinese Population. Gene.

[60] Erion D.M., Ignatova I.D., Yonemitsu S., Nagai Y., Chatterjee P., Weismann D., Hsiao J.J., Zhang D., Iwasaki T., Stark R. (2009). Prevention of hepatic steatosis and hepatic insulin resistance by knockdown of cAMP response element-binding protein. Cell Metab..

[61] Teli D.M., Gajjar A.K. (2023). Glycogen synthase kinase-3: A potential target for diabetes. Bioorg. Med. Chem..

[62] Maqbool M., Hoda N. (2017). GSK3 inhibitors in the therapeutic development of diabetes, cancer and neurodegeneration: Past, present and future. Curr. Pharm. Des..

[63] Wang L., Li J., Di L.J. (2022). Glycogen Synthesis and Beyond: A Comprehensive Review of GSK3 as a Key Regulator of Metabolic Pathways and a Therapeutic Target for Treating Metabolic Diseases. Med. Res. Rev..

[64] Zhang Y., Huang N.Q., Yan F., Sun X., Zheng Z., Yang X., Fang Y., Jiang Y. (2018). Diabetes Mellitus and Alzheimer’s Disease: GSK-3β as a Potential Link. Behav. Brain Res..

[65] Chehimi M., Vidal H., Eljaafari A. (2017). Pathogenic Role of IL-17-Producing Immune Cells in Obesity, and Related Inflammatory Diseases. J. Clin. Med..

[66] Lebovitz H.E., Banerji M.A. (2004). Treatment of insulin resistance in diabetes mellitus. Eur. J. Pharmacol..

[67] Teijeiro A., Garrido A., Ferre A., Perna C., Djouder N. (2021). Inhibition of the IL-17A axis in adipocytes suppresses diet-induced obesity and metabolic disorders in mice. Nat. Metab..

[68] Qiu A.W., Cao X., Zhang W.W., Liu Q.H. (2021). IL-17A is involved in diabetic inflammatory pathogenesis by its receptor IL-17RA. Exp. Biol. Med..

[69] Abdel-Moneim A., Bakery H.H., Allam G. (2018). The Potential Pathogenic Role of IL-17/Th17 Cells in Both Type 1 and Type 2 Diabetes Mellitus. Biomed. Pharmacother..

[70] Shaikh S.B., Prabhakar Bhandary Y. (2020). Effect of curcumin on IL-17A mediated pulmonary AMPK kinase/cyclooxygenase-2 expressions via activation of NFκB in bleomycin-induced acute lung injury in vivo. Int. Immunopharmacol..

[71] Chu W.M. (2013). Tumor Necrosis Factor. Cancer Lett..

[72] de Alvaro C., Teruel T., Hernandez R., Lorenzo M. (2004). Tumor necrosis factor alpha produces insulin resistance in skeletal muscle by activation of inhibitor kappaB kinase in a p38 MAPK-dependent manner. J. Biol. Chem..

[73] Liang H., Yin B., Zhang H., Zhang S., Zeng Q., Wang J., Jiang X., Yuan L., Wang C.Y., Li Z. (2008). Blockade of tumor necrosis factor (TNF) receptor type 1-mediated TNF-alpha signaling protected Wistar rats from diet-induced obesity and insulin resistance. Endocrinology.

[74] da Costa R.M., Neves K.B., Mestriner F.L., Louzada-Junior P., Bruder-Nascimento T., Tostes R.C. (2016). TNF-α Induces Vascular Insulin Resistance via Positive Modulation of PTEN and Decreased Akt/eNOS/NO Signaling in High Fat Diet-Fed Mice. Cardiovasc. Diabetol..

[75] Hartsock A., Nelson W.J. (2008). Adherens and tight junctions: Structure, function and connections to the actin cytoskeleton. Biochim. Biophys. Acta.

[76] Haidari M., Zhang W., Willerson J.T., Dixon R.A. (2014). Disruption of Endothelial Adherens Junctions by High Glucose Is Mediated by Protein Kinase C-β-Dependent Vascular Endothelial Cadherin Tyrosine Phosphorylation. Cardiovasc. Diabetol..

[77] Collares-Buzato C.B., Carvalho C.P. (2022). Is Type 2 Diabetes Mellitus Another Intercellular Junction-Related Disorder?. Exp. Biol. Med..

[78] Wang Y., Liu Q., Kang S.G., Huang K., Tong T. (2021). Dietary Bioactive Ingredients Modulating the cAMP Signaling in Diabetes Treatment. Nutrients.

[79] Vollert S., Kaessner N., Heuser A., Hanauer G., Dieckmann A., Knaack D., Kley H.P., Beume R., Weiss-Haljiti C. (2012). The glucose-lowering effects of the PDE4 inhibitors roflumilast and roflumilast-N-oxide in db/db mice. Diabetologia.

[80] Yang H., Yang L. (2016). Targeting cAMP/PKA Pathway for Glycemic Control and Type 2 Diabetes Therapy. J. Mol. Endocrinol..

[81] Dalle S., Burcelin R., Gourdy P. (2013). Specific actions of GLP-1 receptor agonists and DPP4 inhibitors for the treatment of pancreatic β-cell impairments in type 2 diabetes. Cell Signal..

[82] Miller R.A., Chu Q., Xie J., Foretz M., Viollet B., Birnbaum M.J. (2013). Biguanides suppress hepatic glucagon signalling by decreasing production of cyclic AMP. Nature.

[83] Ookawara M., Nio Y. (2022). Phosphodiesterase 4 inhibitors in diabetic nephropathy. Cell. Signal..

[84] Dehghan F., Haerian B.S., Muniandy S., Yusof A., Dragoo J.L., Salleh N. (2014). The effect of relaxin on the musculoskeletal system. Scand. J. Med. Sci. Sports.

[85] Bonner J.S., Lantier L., Hocking K.M., Kang L., Owolabi M., James F.D., Bracy D.P., Brophy C.M., Wasserman D.H. (2013). Relaxin Treatment Reverses Insulin Resistance in Mice Fed a High-Fat Diet. Diabetes.

[86] Piperi C., Goumenos A., Adamopoulos C., Papavassiliou A.G. (2015). AGE/RAGE signalling regulation by miRNAs: Associations with diabetic complications and therapeutic potential. Int. J. Biochem. Cell Biol..

[87] Taguchi K., Fukami K. (2023). RAGE signaling regulates the progression of diabetic complications. Front. Pharmacol..

[88] Giovannucci E., Harlan D.M., Archer M.C., Bergenstal R.M., Gapstur S.M., Habel L.A., Pollak M., Regensteiner J.G., Yee D. (2010). Diabetes and cancer: A consensus report. Diabetes Care.

[89] Harding J.L., Shaw J.E., Peeters A., Cartensen B., Magliano D.J. (2015). Cancer risk among people with type 1 and type 2 diabetes: Disentangling true associations, detection bias, and reverse causation. Diabetes Care.

[90] Huo Q., Wang J., Zhang N., Xie L., Yu H., Li T. (2022). Editorial: The relationship between diabetes and cancers and its underlying mechanisms. Front. Endocrinol..

[91] Zhu B., Qu S. (2022). The Relationship Between Diabetes Mellitus and Cancers and Its Underlying Mechanisms. Front. Endocrinol..

[92] Rahman I., Athar M.T., Islam M. (2021). Type 2 Diabetes, Obesity, and Cancer Share Some Common and Critical Pathways. Front. Oncol..

[93] Jung U.J., Cho Y.Y., Choi M.S. (2016). Apigenin Ameliorates Dyslipidemia, Hepatic Steatosis and Insulin Resistance by Modulating Metabolic and Transcriptional Profiles in the Liver of High-Fat Diet-Induced Obese Mice. Nutrients.

[94] Kalivarathan J., Kalaivanan K., Chandrasekaran S.P., Nanda D., Ramachandran V., Venkatraman A.C. (2020). Apigenin modulates hippocampal CREB-BDNF signaling in high fat, high fructose diet-fed rats. J. Funct. Foods.

[95] Feng X., Weng D., Zhou F., Owen Y.D., Qin H., Zhao J., Huang Y., Chen J., Fu H., Yang N. (2016). Activation of PPARγ by a Natural Flavonoid Modulator, Apigenin Ameliorates Obesity-Related Inflammation Via Regulation of Macrophage Polarization. EBioMedicine.

[96] Funakoshi-Tago M., Nakamura K., Tago K., Mashino T., Kasahara T. (2011). Anti-inflammatory activity of structurally related flavonoids, Apigenin, Luteolin and Fisetin. Int. Immunopharmacol..

[97] Liu H.J., Fan Y.L., Liao H.H., Liu Y., Chen S., Ma Z.G., Zhang N., Yang Z., Deng W., Tang Q.Z. (2017). Apigenin alleviates STZ-induced diabetic cardiomyopathy. Mol. Cell. Biochem..

[98] Kiraly A.J., Soliman E., Jenkins A., Van Dross R.T. (2016). Apigenin inhibits COX-2, PGE2, and EP1 and also initiates terminal differentiation in the epidermis of tumor bearing mice. Prostaglandins Leukot. Essent. Fat. Acids.

[99] Fan J., Johnson M.H., Lila M.A., Yousef G., de Mejia E.G. (2013). Berry and Citrus Phenolic Compounds Inhibit Dipeptidyl Peptidase IV: Implications in Diabetes Management. Evid.-Based Complement. Altern. Med..

[100] Wang X., Yang J., Li H., Shi S., Peng X. (2022). Mechanistic Study and Synergistic Effect on Inhibition of α-Amylase by Structurally Similar Flavonoids. J. Mol. Liq..

[101] Na B., Nguyen P.H., Zhao B.T., Vo Q.H., Min B.S., Woo M.H. (2016). Protein tyrosine phosphatase 1B (PTP1B) inhibitory activity and glucosidase inhibitory activity of compounds isolated from Agrimonia pilosa. Pharm. Biol..

[102] Chen M., Wang K., Zhang Y., Zhang M., Ma Y., Sun H., Jin Z., Zheng H., Jiang H., Yu P. (2019). New Insights into the Biological Activities of Chrysanthemum morifolium: Natural Flavonoids Alleviate Diabetes by Targeting α-Glucosidase and the PTP-1B Signaling Pathway. Eur. J. Med. Chem..

[103] Wang Y., Wang A., Alkhalidy H., Luo J., Moomaw E., Neilson A.P., Liu D. (2020). Flavone Hispidulin Stimulates Glucagon-Like Peptide-1 Secretion and Ameliorates Hyperglycemia in Streptozotocin-Induced Diabetic Mice. Mol. Nutr. Food Res..

[104] Yu C.I., Cheng C.I., Kang Y.F., Chang P.C., Lin I.P., Kuo Y.H., Jhou A.J., Lin M.Y., Chen C.Y., Lee C.H. (2020). Hispidulin Inhibits Neuroinflammation in Lipopolysaccharide-Activated BV2 Microglia and Attenuates the Activation of Akt, NF-κB, and STAT3 Pathway. Neurotox. Res..

[105] Qin W., Xi J., He B., Zhang B., Luan H., Wu F. (2016). Ameliorative effects of hispidulin on high glucose-mediated endothelial dysfunction via inhibition of PKCβII-associated NLRP3 inflammasome activation and NF-κB signaling in endothelial cells. J. Funct. Foods.

[106] Liang C., Zang J., Ndi C., Semple S.J., Buirchell B., Coriani S., Møller B.L., Staerk D. (2023). Identification of new PTP1B-inhibiting decipiene diterpenoid esters from Eremophila clarkei by high-resolution PTP1B inhibition profiling, enzyme kinetics analysis, and molecular docking. Bioorg. Chem..

[107] Huang D.W., Shen S.C. (2012). Caffeic acid and cinnamic acid ameliorate glucose metabolism via modulating glycogenesis and gluconeogenesis in insulin-resistant mouse hepatocytes. J. Funct. Foods.

[108] Oboh G., Ademosun A.O., Ayeni P.O., Omojokun O.S., Bello F. (2015). Comparative Effect of Quercetin and Rutin on α-Amylase, α-Glucosidase, and Some Pro-Oxidant-Induced Lipid Peroxidation in Rat Pancreas. Comp. Clin. Path..

[109] Istyastono E.P., Yuniarti N., Prasasty V.D., Mungkasi S., Waskitha S.S.W., Yanuar M.R.S., Riswanto F.D.O. (2023). Caffeic Acid in Spent Coffee Grounds as a Dual Inhibitor for MMP-9 and DPP-4 Enzymes. Molecules.

[110] Muthusamy V.S., Saravanababu C., Ramanathan M., Bharathi Raja R., Sudhagar S., Anand S., Lakshmi B.S. (2010). Inhibition of protein tyrosine phosphatase 1B and regulation of insulin signalling markers by caffeoyl derivatives of chicory (Cichorium intybus) salad leaves. Br. J. Nutr..

[111] Fu W., Wang H., Ren X., Yu H., Lei Y., Chen Q. (2017). Neuroprotective effect of three caffeic acid derivatives via ameliorate oxidative stress and enhance PKA/CREB signaling pathway. Behav. Brain Res..

[112] Trang N.T.T., Chiu W.C., Feng Y.T., Hsieh S.L., Tung D.D., Chang J., Fong T.H. (2022). Caffeic Acid Phenethyl Ester Inhibits Basal Lipolysis by Activating PPAR-Gamma and Increasing Lipid Droplet-Associated Perilipin in Mature Rat Adipocytes. Evid. Based Complement. Alternat. Med..

[113] Natarajan K., Singh S., Burke T.R., Grunberger D., Aggarwal B.B. (1996). Caffeic acid phenethyl ester is a potent and specific inhibitor of activation of nuclear transcription factor NF-kappa B. Proc. Natl. Acad. Sci. USA.

[114] Cai H., Huang X., Xu S., Shen H., Zhang P., Huang Y., Jiang J., Sun Y., Jiang B., Wu X. (2016). Discovery of Novel Hybrids of Diaryl-1,2,4-Triazoles and Caffeic Acid as Dual Inhibitors of Cyclooxygenase-2 and 5-Lipoxygenase for Cancer Therapy. Eur. J. Med. Chem..

[115] Choi H.G., Tran P.T., Lee J.H., Min B.S., Kim J.A. (2018). Anti-Inflammatory Activity of Caffeic Acid Derivatives Isolated from the Roots of Salvia miltiorrhiza Bunge. Arch. Pharm. Res..

[116] Wen Y.J., Yin M.C. (2017). The Anti-Inflammatory and Anti-Glycative Effects of Rosmarinic Acid in the Livers of Type 1 Diabetic Mice. BioMedicine.

[117] Ou J., Huang J., Wang M., Ou S. (2017). Effect of Rosmarinic Acid and Carnosic Acid on AGEs Formation in Vitro. Food Chem..

[118] Azhar M.K., Anwar S., Hasan G.M., Shamsi A., Islam A., Parvez S., Hassan M.I. (2023). Comprehensive Insights into Biological Roles of Rosmarinic Acid: Implications in Diabetes, Cancer, and Neurodegenerative Diseases. Nutrients.

[119] Runtuwene J., Cheng K.C., Asakawa A., Amitani H., Amitani M., Morinaga A., Takimoto Y., Kairupan B.H., Inui A. (2016). Rosmarinic acid ameliorates hyperglycemia and insulin sensitivity in diabetic rats, potentially by modulating the expression of PEPCK and GLUT4. Drug Des. Dev. Ther..

[120] Den Hartogh D.J., Vlavcheski F., Tsiani E. (2023). Muscle Cell Insulin Resistance Is Attenuated by Rosmarinic Acid: Elucidating the Mechanisms Involved. Int. J. Mol. Sci..

[121] Han J., Wang D., Ye L., Li P., Hao W., Chen X., Ma J., Wang B., Shang J., Li D. (2017). Rosmarinic Acid Protects against Inflammation and Cardiomyocyte Apoptosis during Myocardial Ischemia/Reperfusion Injury by Activating Peroxisome Proliferator-Activated Receptor Gamma. Front. Pharmacol..

[122] Bower A.M., Real Hernandez L.M., Berhow M.A., de Mejia E.G. (2014). Bioactive Compounds from Culinary Herbs Inhibit a Molecular Target for Type 2 Diabetes Management, Dipeptidyl Peptidase IV. J. Agric. Food Chem..

[123] Salinas-Arellano E., Pérez-Vásquez A., Rivero-Cruz I., Torres-Colin R., González-Andrade M., Rangel-Grimaldo M., Mata R. (2020). Flavonoids and Terpenoids with PTP-1B Inhibitory Properties from the Infusion of Salvia amarissima Ortega. Molecules.

[124] Funke I., Melzig M.F. (2005). Effect of different phenolic compounds on α-amylase activity: Screening by microplate-reader based kinetic assay. Die Pharmazie.

[125] Tshiyoyo K.S., Bester M.J., Serem J.C., Apostolides Z. (2022). In-silico reverse docking and in-vitro studies identified curcumin, 18α-glycyrrhetinic acid, rosmarinic acid, and quercetin as inhibitors of α-glucosidase and pancreatic α-amylase and lipid accumulation in HepG2 cells, important type 2 diabetes targets. J. Mol. Struct..

[126] Teodoro T., Zhang L., Alexander T., Yue J., Vranic M., Volchuk A. (2023). Regulation of glucose metabolism by IL-1β and TNFα in pancreatic β-cells. Endocrinology.

[127] Wang W., Chen K., Xia Y., Mo W., Wang F., Dai W., Niu P. (2018). The Hepatoprotection by Oleanolic Acid Preconditioning: Focusing on PPARα Activation. PPAR Res..

[128] Loza-Rodríguez H., Estrada-Soto S., Alarcón-Aguilar F.J., Huang F., Aquino-Jarquín G., Fortis-Barrera Á., Giacoman-Martínez A., Almanza-Pérez J.C. (2020). Oleanolic acid induces a dual agonist action on PPARγ/α and GLUT4 translocation: A pentacyclic triterpene for dyslipidemia and type 2 diabetes. Eur. J. Pharmacol..

[129] Iskender H., Dokumacioglu E., Hayirli A., Kapakin K.A.T., Bolat I., Kirman E.M. (2023). Effects of oleanolic acid administration on renal NF-kB, IL-18, IL-6, YKL-40, and KIM-1 in experimental diabetic rats. Iran. J. Basic Med. Sci..

[130] Zhang S.Q., Lin K.L., Law C.Y., Liu B., Fu X.Q., Tse W.S., Wong S.S.M., Sze S.C.W., Yung K.K.L. (2018). Oleanolic Acid Enhances Neural Stem Cell Migration, Proliferation, and Differentiation in Vitro by Inhibiting GSK3β Activity. Cell Death Discov..

[131] Zhang Y.N., Zhang W., Hong D., Shi L., Shen Q., Li J.Y., Li J., Hu L.H. (2008). Oleanolic Acid and Its Derivatives: New Inhibitor of Protein Tyrosine Phosphatase 1B with Cellular Activities. Bioorg. Med. Chem..

[132] Ramírez-Espinosa J.J., Rios M.Y., Paoli P., Flores-Morales V., Camici G., de la Rosa-Lugo V., Hidalgo-Figueroa S., Navarrete-Vázquez G., Estrada-Soto S. (2014). Synthesis of oleanolic acid derivatives: In vitro, in vivo and in silico studies for PTP-1B inhibition. Eur. J. Med. Chem..

[133] Raoufi S., Baluchnejadmojarad T., Roghani M., Ghazanfari T., Khojasteh F., Mansouri M. (2015). Antidiabetic potential of salvianolic acid B in multiple low-dose streptozotocin-induced diabetes. Pharm. Biol..

[134] Shi Y., Pan D., Yan L., Chen H., Zhang X., Yuan J., Mu B. (2020). Salvianolic acid B improved insulin resistance through suppression of hepatic ER stress in ob/ob mice. Biochem. Biophys. Res. Commun..

[135] Pan Y., Zhao W., Zhao D., Wang C., Yu N., An T., Mo F., Liu J., Miao J., Lv B. (2018). Salvianolic Acid B Improves Mitochondrial Function in 3T3-L1 Adipocytes Through a Pathway Involving PPARγ Coactivator-1α (PGC-1α). Front. Pharmacol..

[136] Wang P., Xu S., Li W., Wang F., Yang Z., Jiang L., Wang Q., Huang M., Zhou P. (2014). Salvianolic Acid B Inhibited PPARγ Expression and Attenuated Weight Gain in Mice with High-Fat Diet-Induced Obesity. Cell. Physiol. Biochem..

[137] Chen Y.L., Hu C.S., Lin F.Y., Chen Y.H., Sheu L.M., Ku H.H., Shiao M.S., Chen J.W., Lin S.J. (2006). Salvianolic Acid B Attenuates Cyclooxygenase-2 Expression in Vitro in LPS-Treated Human Aortic Smooth Muscle Cells and In Vivo in the Apolipoprotein-E-Deficient Mouse Aorta. J. Cell. Biochem..

[138] Bai X., Fan W., Luo Y., Liu Y., Zhang Y., Liao X. (2022). Fast Screening of Protein Tyrosine Phosphatase 1B Inhibitor from Salvia miltiorrhiza Bge by Cell Display-Based Ligand Fishing. Molecules.

[139] Paudel P., Seong S.H., Zhou Y., Park C.H., Yokozawa T., Jung H.A., Choi J.S. (2018). Rosmarinic Acid Derivatives’ Inhibition of Glycogen Synthase Kinase-3β Is the Pharmacological Basis of Kangen-Karyu in Alzheimer’s Disease. Molecules.

[140] Wang B., Sun J., Shi Y., Le G. (2017). Salvianolic Acid B Inhibits High-Fat Diet-Induced Inflammation by Activating the Nrf2 Pathway. J. Food Sci..

[141] Agu P.C., Afiukwa C.A., Orji O.U., Ezeh E.M., Ofoke I.H., Ogbu C.O., Ugwuja E.I., Aja P.M. (2023). Molecular Docking as a Tool for the Discovery of Molecular Targets of Nutraceuticals in Disease Management. Sci. Rep..

[142] Meng X.Y., Zhang H.X., Mezei M., Cui M. (2011). Molecular docking: A powerful approach for structure-based drug discovery. Curr. Comput.-Aided Drug Des..

[143] Brayer G.D., Sidhu G., Maurus R., Rydberg E.H., Braun C., Wang Y., Nguyen N.T., Overall C.M., Withers S.G. (2000). Subsite Mapping of the Human Pancreatic Alpha-Amylase Active Site Through Structural, Kinetic, and Mutagenesis Techniques. Biochemistry.

[144] Maurus R., Begum A., Williams L.K., Fredriksen J.R., Zhang R., Withers S.G., Brayer G.D. (2008). Alternative catalytic anions differentially modulate human alpha-amylase activity and specificity. Biochemistry.

[145] Krishnan N., Krishnan K., Connors C.R., Choy M.S., Page R., Peti W., Van Aelst L., Shea S.D., Tonks N.K. (2015). PTP1B inhibition suggests a therapeutic strategy for Rett syndrome. J. Clin. Investig..

[146] Sun J.P., Fedorov A.A., Lee S.Y., Guo X.L., Shen K., Lawrence D.S., Almo S.C., Zhang Z.Y. (2003). Crystal structure of PTP1B complexed with a potent and selective bidentate inhibitor. J. Biol. Chem..

[147] Kirby M., Yu D.M., O’Connor S., Gorrell M.D. (2009). Inhibitor selectivity in the clinical application of dipeptidyl peptidase-4 inhibition. Clin. Sci..

[148] Pissarnitski D.A., Zhao Z., Cole D., Wu W.L., Domalski M., Clader J.W., Scapin G., Voigt J., Soriano A., Kelly T. (2016). Scaffold-hopping from xanthines to tricyclic guanines: A case study of dipeptidyl peptidase 4 (DPP4) inhibitors. Bioorg. Med. Chem..

[149] Gentile G., Merlo G., Pozzan A., Bernasconi G., Bax B., Bamborough P., Bridges A., Carter P., Neu M., Yao G. (2012). 5-Aryl-4-carboxamide-1,3-oxazoles: Potent and selective GSK-3 inhibitors. Bioorg. Med. Chem. Lett..

[150] Pandey M.K., DeGrado T.R. (2016). Glycogen Synthase Kinase-3 (GSK-3)-Targeted Therapy and Imaging. Theranostics.

[151] Pan D.S., Wang W., Liu N.S., Yang Q.J., Zhang K., Zhu J.Z., Shan S., Li Z.B., Ning Z.Q., Huang L. (2017). Chiglitazar Preferentially Regulates Gene Expression via Configuration-Restricted Binding and Phosphorylation Inhibition of PPARγ. PPAR Res..

[152] Lee M.A., Tan L., Yang H., Im Y.G., Im Y.J. (2017). Structures of PPARγ complexed with lobeglitazone and pioglitazone reveal key determinants for the recognition of antidiabetic drugs. Sci. Rep..

[153] Orlando B.J., Malkowski M.G. (2016). Substrate-selective Inhibition of Cyclooxygenase-2 by Fenamic Acid Derivatives Is Dependent on Peroxide Tone. J. Biol. Chem..

[154] Rouzer C.A., Marnett L.J. (2020). Structural and Chemical Biology of the Interaction of Cyclooxygenase with Substrates and Non-Steroidal Anti-Inflammatory Drugs. Chem. Rev..

[155] Jacobs M.D., Harrison S.C. (1998). Structure of an IkappaBalpha/NF-kappaB complex. Cell.

[156] Escalante C.R., Shen L., Thanos D., Aggarwal A.K. (2002). Structure of NF-kappaB p50/p65 heterodimer bound to the PRDII DNA element from the interferon-beta promoter. Structure.

[157] Daina A., Michielin O., Zoete V. (2017). SwissADME: A Free Web Tool to Evaluate Pharmacokinetics, Drug-Likeness, and Medicinal Chemistry Friendliness of Small Molecules. Sci. Rep..

[158] Ugwor E.I., James A.S., Amuzat A.I., Ezenandu E.O., Ugbaja V.C., Ugbaja R.N. (2022). Network pharmacology-based elucidation of bioactive compounds in propolis and putative underlying mechanisms against type-2 diabetes mellitus. Pharmacol. Res.-Mod. Chin. Med..

[159] Patil V.S., Harish D.R., Vetrivel U., Roy S., Deshpande S.H., Hegde H.V. (2022). Hepatitis C Virus NS3/4A Inhibition and Host Immunomodulation by Tannins from Terminalia chebula: A Structural Perspective. Molecules.

[160] Sun J., Liu B., Wang R., Yuan Y., Wang J., Zhang L. (2022). Computation-Based Discovery of Potential Targets for Rheumatoid Arthritis and Related Molecular Screening and Mechanism Analysis of Traditional Chinese Medicine. Dis. Markers.

[161] Alamri M.A. (2023). Bioinformatics and Network Pharmacology-Based Study to Elucidate the Multi-Target Pharmacological Mechanism of the Indigenous Plants of Medina Valley in Treating HCV-Related Hepatocellular Carcinoma. Saudi Pharm. J..

[162] El-Atawneh S., Goldblum A. (2023). Activity Models of Key GPCR Families in the Central Nervous System: A Tool for Many Purposes. J. Chem. Inf. Model..

[163] Zhou D.Y., Mou X., Liu K., Liu W.H., Xu Y.Q., Zhou D. (2020). In Silico Prediction and Validation of Potential Therapeutic Genes in Pancreatic β-Cells Associated with Type 2 Diabetes. Exp. Ther. Med..

[164] Mukherjee A., Yadav P.H., Mukunthan K.S. (2023). Unveiling potential targeted therapeutic opportunities for co-overexpressed targeting protein for Xklp2 and Aurora-A kinase in lung adenocarcinoma. Mol. Biotechnol..

[165] Ononamadu C.J., Abdalla M., Ihegboro G.O., Li J., Owolarafe T.A., John T.D., Tian Q. (2021). In silico identification and study of potential anti-mosquito juvenile hormone binding protein (MJHBP) compounds as candidates for dengue virus—Vector insecticides. Biochem. Biophys. Rep..

[166] Ramírez D., Caballero J. (2018). Is It Reliable to Take the Molecular Docking Top Scoring Position as the Best Solution without Considering Available Structural Data?. Molecules.

[167] Galli C.L., Sensi C., Fumagalli A., Parravicini C., Marinovich M., Eberini I. (2014). A computational approach to evaluate the androgenic affinity of iprodione, procymidone, vinclozolin and their metabolites. PLoS ONE.

[168] Nissink J.W. (2009). Simple size-independent measure of ligand efficiency. J. Chem. Inf. Model..

